# Problematic smartphone use and academic procrastination over an academic year: a four-wave random intercept cross-lagged panel study of Chinese undergraduates

**DOI:** 10.3389/fpsyg.2026.1935647

**Published:** 2026-10-06

**Authors:** Peng Che

**Affiliations:** Zhejiang University of Finance and Economics, Hangzhou, China

**Keywords:** academic procrastination, Chinese undergraduates, longitudinal, problematic smartphone use, random intercept cross-lagged panel model, self-regulation

## Abstract

Problematic smartphone use (PSU) and academic procrastination (AP) are reliably correlated among university students, but the evidence base is almost entirely cross-sectional, and the only existing longitudinal study tested a single direction using a model that cannot separate stable trait-like co-occurrence from genuine within-person dynamics. This study used a four-wave Random Intercept Cross-Lagged Panel Model (RI-CLPM) to test both directions of within-person prediction simultaneously in a sample of Chinese undergraduates (*N* = 512 at intake, 422 retained by the fourth wave) surveyed across a full academic year, and to test whether the well-documented PSU-AP association persists after accounting for trait self-control and trait negative affect. The model fit the data well, χ^2^(45) = 67.72, CFI = 0.994, RMSEA = 0.031, SRMR = 0.022. Within-person increases in PSU predicted later increases in AP in one interval only, the final T3 → T4 interval (β = 0.374, *p* < 0.001); the two earlier intervals were non-significant, and a stationarity test confirmed that the three are not statistically equivalent, Δχ^2^(2) = 11.16, *p* = 0.004. The pooled estimate, γ = 0.213, is accordingly reported as a descriptive summary of a heterogeneous set of paths rather than as a stable within-person effect, and the wave-specific estimates, the T3 → T4 path in particular, carry the evidence for H1. Within-person increases in AP were not detected as predictors of later PSU at any wave (pooled δ = −0.013, *p* = 0.863, a defensible single estimate since these three waves were statistically equivalent); a design-stage Monte Carlo check gave about 0.81 power for an effect of the hypothesized size, so this is a reasonably informative null: the AP → PSU pathway was not detected at a three-month lag in this sample, which is not the same as showing it absent. The two directions differed in estimated magnitude; an equality-constraint likelihood-ratio test within the RI-CLPM confirmed the asymmetry at the final interval, Δχ^2^(1) = 9.16, *p* = 0.002, although the γ side rests on a single interval, so the asymmetry is specific to the T3 → T4 window. The random-intercept correlation between PSU and AP remained significant in the full model adjusting for all six T1 covariates (r = 0.250, *p* < 0.001), indicating a substantial trait-level component. However, the pooled cross-lagged estimates did not hold their values once a common-method factor was added. That specification is too weakly identified to re-estimate the correlation at all, so shared-method inflation cannot be excluded for either. A traditional cross-lagged panel model without this trait/state decomposition showed markedly inflated stability and cross-lagged estimates by comparison. PSU met metric but not scalar longitudinal invariance, which qualifies the interpretation of within-person change on that construct. However, refitting the model on an item-reduced PSU composite left the focal estimate essentially unchanged (T3 → T4 β = 0.378). These findings indicate that the PSU–AP relationship, as observed here, is interval-specific rather than uniform, directionally asymmetric within the one interval in which a within-person effect was detected, and only partially dynamic; they are best read as hypotheses about how future intervention research might be sequenced and targeted rather than as direct guidance for practice.

## 1 Introduction

Problematic smartphone use (PSU; compulsive, poorly regulated phone engagement that interferes with daily functioning, Kwon et al., 2013) and academic procrastination (AP; the voluntary, irrational delay of an intended task despite anticipated costs, Steel, 2007) are two of the most widely studied correlates of underperformance in higher education. Wherever researchers have measured the two constructs together, they are reliably and positively associated: a 2024 meta-analysis of more than 20 studies found the pooled association to be of a small-to-moderate magnitude (Chen and Lyu, 2024). Both behaviors carry independent academic costs, lower grades, slower degree progress, and elevated dropout risk. They are already treated as connected problems in practice, often without evidence about which one, if either, drives the other over time. The existing evidence base cannot say anything about direction, sequence, or the difference between a stable trait and a moving state, and that gap sits at the center of the present study.

This gap is structural, not a peculiarity of the two behaviors. Almost the entire PSU–AP literature is cross-sectional, and the single longitudinal exception (reviewed in Section 2) tested only one direction and did not separate a student’s stable trait-like standing from wave-to-wave fluctuation. That distinction is not a minor statistical nicety: methodological work since Hamaker et al. (2015) shows that models blind to it can produce cross-lagged effects that are merely stable between-person differences in disguise, or mask genuine within-person dynamics entirely.

The Random Intercept Cross-Lagged Panel Model (RI-CLPM) resolves this conflation by partitioning each wave’s score into a person-specific stable component and a wave-specific deviation from that person’s own trajectory. It has already been applied productively to adjacent problems, PSU alongside loneliness and depression, academic self-concept alongside achievement, and body dissatisfaction across adolescence, since Hamaker et al.’s critique. It has not yet been applied to PSU and AP, despite an executive-control account of problematic technology use and an emotion-regulation account of procrastination, each implying a specific, opposite-facing pathway between the two behaviors (reviewed in Section 2). Neither literature has been brought into contact with the other in a design capable of testing both directions and both the between- and within-person components at once.

This study does that. Using a four-wave RI-CLPM fitted across a full academic year of data from Chinese undergraduates (target *N* = 512 at intake), it asks whether within-person increases in PSU precede later within-person increases in AP (H1), whether the reverse also holds (H2), and whether the well-documented PSU–AP correlation is substantially a stable, trait-level phenomenon that survives statistical control for trait self-control and trait negative affect (H3), alongside an exploratory question of whether one directional pathway outweighs the other (RQ1). To our knowledge, based on a search of the OpenAlex index, no published study has applied a random-intercept panel technique to this construct pairing. Accordingly, the contribution is not a new theory but a needed correction to how an already-theorized claim, that PSU and AP feed each other over a term, has actually been tested. Separating that claim from its simpler alternative, that PSU and AP merely coexist in the same kinds of students, bears directly on whether a phone-use intervention, a procrastination-focused intervention, or one targeting the shared underlying disposition is the more defensible next step.

Section 2 reviews the I-PACE and self-regulatory-failure/TST literatures, situates the design against the closest prior work, and develops the four hypotheses above; Section 3 describes the sample, measures, and analytic strategy; Section 4 reports the results; and Section 5 discusses their theoretical and practical implications, limitations, and directions for future work.

## 2 Literature review and hypothesis development

This section reviews the two focal constructs in turn, then argues that the existing PSU–procrastination literature has a specific, remediable design problem, not a theoretical one, and closes by developing four hypotheses that follow from reading I-PACE and self-regulatory accounts of procrastination as complementary rather than competing frameworks.

### 2.1 Problematic smartphone use: conceptualization and the I-PACE model

PSU refers to a pattern of phone engagement characterized by loss of control, salience, and interference with daily obligations, most commonly operationalized with Kwon et al.'s (2013) Smartphone Addiction Scale–Short Version (SAS-SV). Whether PSU constitutes a genuine behavioral addiction, a maladaptive habit, or the tail end of normal usage variation remains contested (Panova and Carbonell, 2018; Montag et al., 2019), and self-report measures correlate only modestly with logged, objective usage data (Ellis et al., 2019; Parry et al., 2021). This study does not resolve that debate: the research question concerns what problematic-use scores predict about a student’s subsequent academic functioning, regardless of where PSU ultimately sits on the taxonomy of behavioral addictions.

The dominant etiological account of PSU is the Interaction of Person-Affect-Cognition-Execution (I-PACE) model (Brand et al., 2016, 2019, 2025), developed for internet-use disorders and extended to smartphone use. I-PACE describes a cyclical process: person-level predispositions (impulsivity, affective vulnerability, weak trait self-control) shape affective and cognitive responses to situational cues (craving, coping motives, attentional bias toward the device), which draw on and gradually deplete executive control resources. I-PACE treats reduced executive control as both a cause and a consequence of problematic use: early use is motivated by coping and reward, but repeated engagement erodes the inhibitory capacity that would otherwise regulate future use, producing a self-sustaining cycle rather than a one-off choice. Most empirical tests of I-PACE’s downstream consequences have targeted mental-health outcomes (loneliness, depressive symptoms, general distress; Shi et al., 2023; Zhang et al., 2023; Zhao et al., 2024). Recent I-PACE-based work has extended the same person–affect–cognition logic to short-video dependence among Chinese university students and traced serial cognitive and affective pathways from core personality traits to problematic use (Zhang et al., 2026), and comparatively little work has asked whether the same erosion appears in an academic, achievement-relevant outcome instead.

### 2.2 Academic procrastination: self-regulatory failure and temporal self-regulation theory

AP is the voluntary, ultimately irrational delay of an intended academic task despite the delayer’s own expectation of being worse off for it (Steel, 2007), with well-documented costs for academic performance (Kim and Seo, 2015). Steel’s meta-analysis remains the field’s anchor, framing procrastination principally as a self-regulatory failure (Pychyl and Flett, 2012): low task-related self-efficacy, task aversiveness, and, most relevant here, impulsiveness and weak self-control predict who procrastinates and how often. Temporal Self-Regulation Theory (TST; Hall and Fong, 2007, 2010) extends this logic by modeling behavior as a joint function of intention, habit, and self-regulatory capacity, predicting that even a well-intentioned student will fail to act when momentary regulatory capacity cannot override a more immediately available, rewarding alternative, consistent with evidence that self-regulated learning strategies and procrastination are closely, inversely linked (Wolters et al., 2017). A complementary strand treats procrastination not merely as a capacity failure but as an active, maladaptive emotion-regulation strategy: delaying an aversive task provides short-term relief from the negative affect it provokes, at the cost of compounding guilt, anxiety, and further aversiveness later (Sirois and Pychyl, 2013; Sirois, 2014, 2023). This mood-repair account matters here because, unlike a pure capacity-depletion story, it predicts what a procrastinating student does next: not nothing, but something rewarding and low-effort, exactly the profile of a short smartphone session.

Measurement in this literature is more fragmented than the theory: at least four validated short-form procrastination scales are in active use (Yockey, 2016; Özer et al., 2013; Svartdal and Steel, 2017), differing in item count, target behavior, and factor structure. This matters for a repeated-measures design: a longer instrument accumulates more respondent burden across four administrations, whereas a shorter one has less item-level redundancy if full longitudinal invariance is not supported, a tradeoff addressed in the Method section’s choice of instrument.

### 2.3 The PSU–academic procrastination literature: cross-sectional consensus, longitudinal silence

Both constructs, considered separately, already have well-developed longitudinal literatures. PSU has been tracked across two and three waves alongside sleep quality, depressive symptoms, loneliness, and rumination, generally via cross-lagged or RI-CLPM designs in Chinese and Taiwanese student cohorts (Cui et al., 2021; Zhang et al., 2023; Li G. et al., 2023; Shi et al., 2023; Wang et al., 2022; Chen et al., 2023). AP has its own smaller longitudinal literature, concentrated in German, Austrian, and Canadian samples, linking procrastination reciprocally to dropout intentions, study satisfaction, and academic emotions (Scheunemann et al., 2021; Rahimi et al., 2023; Gadosey et al., 2023; Lindner et al., 2023; Kljajić and Gaudreau, 2018). What is missing is any longitudinal traffic between the two constructs, despite a large cross-sectional literature asserting they are related: more than twenty studies report a positive PSU-AP association (Yang et al., 2018; Rozgonjuk et al., 2018; Li et al., 2020; Liu et al., 2022; Tian et al., 2021; Song et al., 2025), synthesized in a 2024 meta-analysis at a small-to-moderate pooled magnitude (Chen and Lyu, 2024), with a comparably sized association reported for the adjacent construct of academic burnout (Li S. et al., 2023). A cross-sectional correlation of this kind is compatible with at least three processes: PSU causing later AP, AP causing later PSU, or neither, with both instead reflecting a shared, stable disposition (e.g., trait self-control) that elevates both behaviors in the same students, and cross-sectional data cannot adjudicate between them. To date, the field has treated the reliable correlation as if it settled the question of a dynamic, reciprocal relationship.

Exactly one study moves beyond this. Hong et al. (2021) followed Chinese adolescents longitudinally. They found that AP predicted later problematic mobile phone use, mediated by a third variable, the first genuine temporal-precedence evidence in this pairing. Two features leave the present question open: it tested only the AP → PSU direction, so whether PSU also predicts later AP, the direction I-PACE’s executive-control account implies, has never been examined; and, like the cross-sectional literature it improves on, it did not separate each adolescent’s stable trait-like standing from wave-to-wave fluctuation, leaving open whether its one confirmed direction reflects a genuine within-person dynamic or partly reflects between-person confounding.

A small number of additional studies identified through the same OpenAlex search plausibly bear on this pairing but could not be confirmed as longitudinal from title/abstract metadata alone; none report a random-intercept design, so their inclusion or exclusion does not change the substantive gap. That search was a scoping check of an index rather than a systematic review: it was not conducted against a pre-registered protocol, no screening log was retained, and no completeness claim is made for it. The novelty claim above should be read as a statement about what we were able to find rather than as the output of a reproducible search procedure. To our knowledge, no published study has used a four-wave RI-CLPM to disentangle between-person trait-like co-occurrence from within-person, bidirectional temporal precedence between PSU and AP in a Chinese undergraduate population. This study addresses that gap, building on Hong et al. (2021) as the closest prior design and on Chen and Lyu's (2024) meta-analysis as the clearest statement of how cross-sectional the existing evidence base otherwise is.

### 2.4 Disentangling trait from state: the case for the random intercept cross-lagged panel model

This point is not specific to Hong et al. (2021); it applies to the traditional cross-lagged panel model (CLPM) generally, wherever it has been used to argue for reciprocal causation between repeatedly measured constructs. Hamaker et al. (2015) showed formally that CLPM cross-lagged coefficients conflate a between-person component (are people chronically high on X also chronically high on Y?) with a within-person component (does this person’s own increase in X precede their own later increase in Y?), which can point in opposite directions or produce spurious cross-lagged effects when trait-like stability is present but unmodeled, the expected case for constructs as dispositionally loaded as PSU and AP. The RI-CLPM resolves this by explicitly modeling a random intercept for each construct (each person’s stable average level across waves) and estimating autoregressive and cross-lagged paths only on the wave-specific deviations from that trait level (Hamaker et al., 2015; Mulder and Hamaker, 2020; Usami, 2020). The model has its own debates: work has questioned whether its between-person component can be partly illusory under certain misspecifications (Lüdtke and Robitzsch, 2021; Robitzsch and Lüdtke, 2024), proposed alternative parameterizations (Andersen, 2021), flagged small-sample instability with fewer than four waves (Zheng and Valente, 2022), and supplied power-analysis tools (Mulder, 2022) and cross-lagged effect-size benchmarks (Orth et al., 2020, 2022). The small-sample point bears directly on design here: RI-CLPM is technically identified with three waves, but a three-wave model estimates only one autoregressive and cross-lagged path per construct and leans more heavily on the random-intercept variance components, one concrete reason for adding a fourth wave (elaborated in the Method section). None of this argues for reverting to standard CLPM when trait-like stability is plausible; it argues for using RI-CLPM carefully, with adequate waves and transparent reporting, the approach adopted here. RI-CLPM has already proven useful for this kind of disentangling problem in adjacent educational-psychology constructs, from academic self-concept and achievement (Burns et al., 2019; Marsh et al., 2022) to teacher–student relationships (Li, 2022), self-control in problematic gaming (Qi et al., 2024), and four-wave Chinese-sample designs specifically (Ren et al., 2023). Applying it to PSU and AP extends this template to a pairing it has not yet reached.

### 2.5 A shared self-regulatory logic, two distinct directional mechanisms

I-PACE and the self-regulatory/emotion-regulation account of procrastination are treated here as complementary lenses rather than a single fused mechanism. Both nonetheless converge on a general-purpose executive/self-regulatory capacity that PSU and AP each draw on and, in different ways, erode. Treating this capacity as domain-general is not arbitrary: trait self-control of this kind independently predicts outcomes as varied as academic grades, impulse control, and relationship quality within the same individuals (Tangney et al., 2004). That evidence concerns a stable trait predicting across domains, though, not whether a state-level dip in regulatory capacity from an evening of phone use carries over into next week’s academic behavior at a three-month grain; this study’s design tests only the latter, more specific claim. Whether heavy phone use draws down the identical resource that timely academic work would otherwise draw on, a correlated but distinct resource, or something else entirely is an open question this design cannot close on its own (Section 5 returns to it). I-PACE’s account of use progressively weakening inhibitory control implies a capacity-erosion pathway: a student whose smartphone engagement climbs in a given period should have measurably less regulatory capacity left for timely academic work in the period that follows, net of how procrastination-prone that student already tends to be. This pathway has a plausible amplifier in a Chinese undergraduate population, where lecture-time social apps, short-video platforms, and messaging are woven through the student day, making a momentary loss of executive control to the device a proximal, everyday candidate driver of subsequent task delay.

*H1:* Within-person increases in PSU at wave t will positively predict within-person increases in AP at wave t + 1, net of AP’s own within-person stability and of stable between-person trait differences in both constructs.

The complementary, emotion-regulation account of procrastination implies the reverse-direction pathway: if procrastination functions partly as a maladaptive mood-repair strategy, a student whose procrastination climbs in a given period should show elevated smartphone engagement in the period that follows, because the negative affect generated by delaying motivates exactly the kind of low-effort, immediately available escape a smartphone provides. Hong et al.'s (2021) finding that procrastination in Chinese adolescents preceded later problematic phone use is direct, if not RI-CLPM-based, precedent for this pathway, and the same contextual point applies in reverse: for a Chinese undergraduate with a smartphone in easy reach at essentially all times, the phone is a low-cost, immediately available mood-repair option after a bout of procrastination-induced guilt or anxiety.

*H2:* Within-person increases in AP at wave *t* will positively predict within-person increases in PSU at wave *t* + 1, net of PSU’s own within-person stability and of stable between-person trait differences in both constructs.

Neither hypothesis claims a discrete mediating mechanism; each is calibrated to what a four-wave RI-CLPM can establish: temporally ordered, within-person predictive association net of trait-level confounding, not a demonstrated causal mechanism.

### 2.6 Between-person confounding: a shared dispositional vulnerability

The within-person pathways above are only half of what a trait-versus-state design can test. Both I-PACE and the self-regulatory-failure account of procrastination independently point to the same class of stable, person-level vulnerabilities, low trait self-control chief among them, alongside general affective vulnerability such as elevated trait depression/anxiety, as common antecedents of chronically elevated PSU and AP alike, apart from any wave-to-wave dynamic traffic between them. If this dispositional account is right, at least part of the well-replicated cross-sectional PSU–AP correlation (Chen and Lyu, 2024) should reside in this shared, stable component, the random-intercept correlation a RI-CLPM estimates directly, rather than in any genuine reciprocal process. This study operationalizes that logic by entering trait self-control and trait negative affect as measured, time-invariant covariates on both random intercepts: full attenuation of the intercept correlation would support the shared-vulnerability account only in its most reducible form, whereas a significant residual correlation would indicate a broader, not-fully-measured shared vulnerability.

*H3:* The random intercept factors for PSU and AP will remain significantly and positively correlated even after statistically controlling for trait self-control and trait negative affect as covariates on both random intercepts.

### 2.7 An open question about directional asymmetry

I-PACE’s emphasis on an escalating, habituating use pattern could suggest that, over a full academic year, the capacity-erosion pathway (H1) should eventually outweigh the more episodic, affect-driven coping pathway (H2). This reading is plausible but not confident: I-PACE’s escalation logic was developed to describe clinical or near-clinical addiction severity, not the largely sub-clinical PSU range expected in a general undergraduate sample, and no study identified here directly compares the relative magnitude of these two directions. Rather than force a prediction the theory does not clearly license, this study treats the comparison as an open empirical question.

*RQ1:* Do the within-person PSU → AP (H1) and AP → PSU (H2) cross-lagged coefficients differ significantly in magnitude, tested via a Wald test of parameter equality across the pooled wave-specific estimates?

Figure 1 summarizes this full hypothesis system as a single conceptual diagram: the between-person random-intercept correlation (H3) at the top, and the within-person autoregressive and cross-lagged structure (H1, H2) below it.

**Figure 1:**
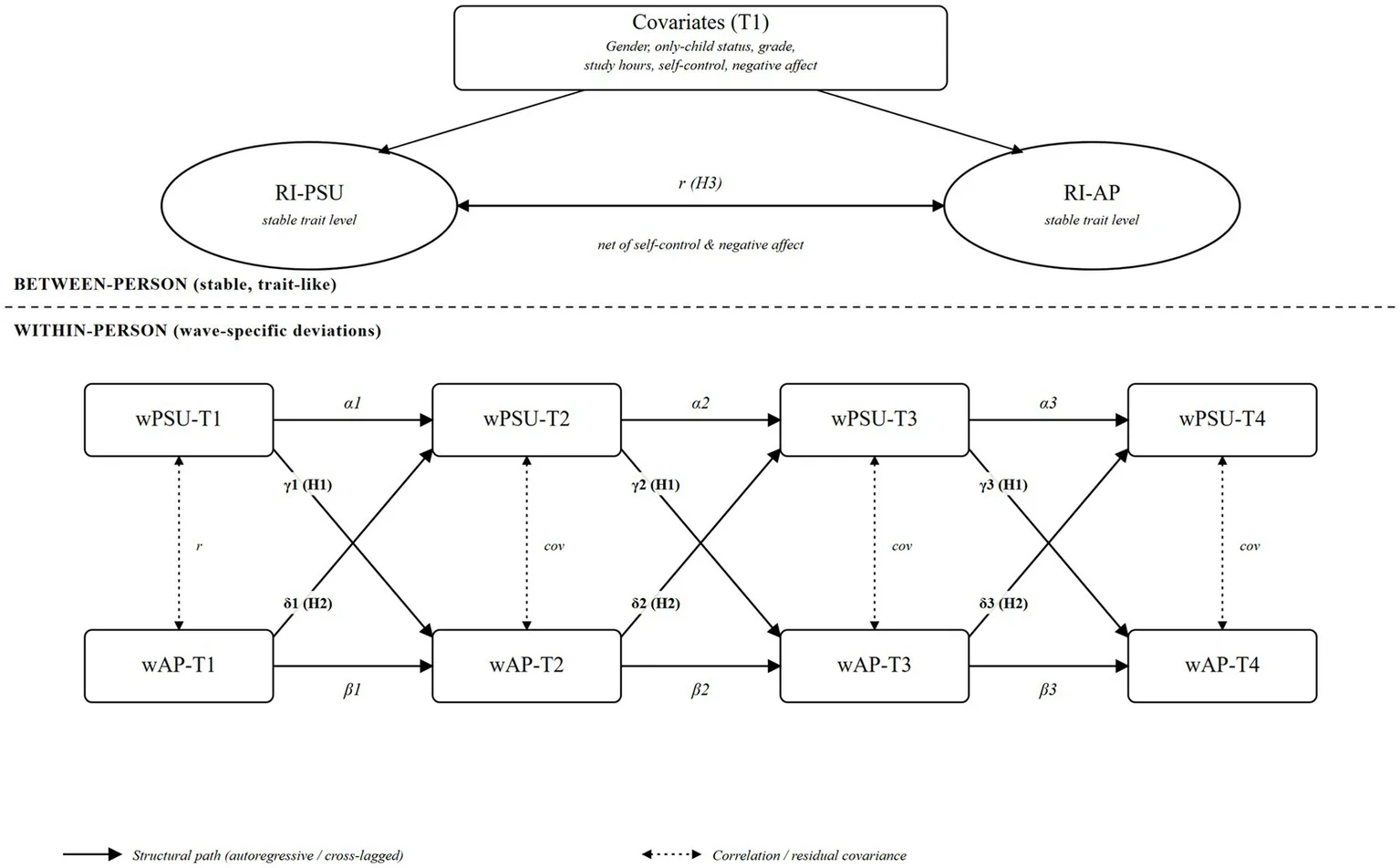
Conceptual RI-CLPM framework. Between-person random intercepts (RI-PSU, RI-AP) are correlated (H3) net of covariates; within-person wave-specific deviations follow autoregressive (α, β) and cross-lagged (γ = H1, δ = H2) paths, with residual covariances at each wave.

## 3 Method

### 3.1 Design and procedure

This study used a four-wave longitudinal panel design spanning one full academic year, with assessments spaced roughly three months apart: T1 in the second or third week of the Fall semester, T2 at the end of the Fall semester (pre-exam week), T3 at the midpoint of the Spring semester, and T4 at the end of the Spring semester (pre-exam week). This spacing aligns each wave with a distinct point in the Chinese undergraduate academic calendar rather than reflecting a validated causal window for PSU–procrastination spillover; no such window has been established in this literature. Because these anchors are calendar-driven, the three inter-wave intervals are not equivalent in content: T1 → T2 and T3 → T4 each fall wholly within one semester and end in a pre-examination period, whereas T2 → T3 spans the Fall examination period, the winter vacation, and the start of a new semester. Exact administration windows were T1 September 25–29, 2023; T2 December 18–22, 2023; T3 March 25–29, 2024; and T4 June 17–21, 2024, giving realized lags of approximately 12, 14, and 12 weeks. This structural difference between intervals directly affects the interval-specific cross-lagged results reported in Section 4.3 and is addressed in Sections 5.1 and 5.4.

### 3.2 Participants and sampling

Participants were undergraduate students at a large, comprehensive Chinese university, recruited via cluster sampling of introductory-level course sections across multiple faculties and majors to avoid a single-classroom or single-major bias. Participation was voluntary, administered through a campus-wide online survey platform, with a small per-wave incentive (a mobile-data top-up, ~10 RMB) to support retention across four administrations. The target intake sample was *N* = 512 at T1. Based on retention patterns for comparable four-wave Chinese panels (e.g., Ren et al., 2023), attrition of roughly 12–15% per interval was anticipated, yielding an expected analytic sample of ~420–450 by T4; participants with data from at least one wave were retained via Full Information Maximum Likelihood (FIML) rather than excluded through listwise deletion. Recruitment covered 14 course sections across 5 faculties. Section identifiers were used to administer the survey but were not carried into the analytic dataset, so no section-level variable, and no proxy for one, is available for a cluster-robust or multilevel robustness check; the consequences are set out in Section 5.4. Age and academic discipline were not recorded at the individual level and cannot be reported, and recruitment at a single institution bounds generalization from this sample in ways set out in Section 5.4. The realized T1 sample comprised 265 men and 247 women; 233 participants (45.5%) were only children; year of study was distributed as 130 (Year 1), 123 (Year 2), 120 (Year 3), and 139 (Year 4); and self-reported weekly self-study hours averaged 18.05 (SD = 6.03). No exclusion criteria beyond the eligibility requirements stated below were applied, and no enrolled participant was removed from the analytic sample.

Sample-size adequacy drew on two sources. First, Mulder's (2022) power-analysis guidance for RI-CLPM (powRICLPM framework) indicates a sample in the 400–500 range is generally adequate for detecting small-to-moderate standardized cross-lagged effects (β ≈ 0.10–0.15) across four waves, which we used as a starting benchmark. Second, a Monte Carlo power check simulated repeated four-wave datasets at *N* = 512 under the hypothesized effect sizes and computed empirical power to detect the focal cross-lagged paths at α = 0.05: base autoregressive parameters of approximately 0.46–0.58 for PSU and 0.47–0.54 for AP, cross-lagged γ of approximately 0.36–0.42, and cross-lagged δ of approximately 0.15–0.21, each with a small independent draw per wave. These population values were fixed at the data-generation stage, not tuned to this study’s final composite-level results; the power statement below is accordingly an *a priori*, design-stage check, not observed power computed after the fact.

Beyond FIML, which assumes data are missing at random (MAR) conditional on observed variables, two further steps addressed attrition: a completer-versus-attriter comparison on all T1 study variables and demographics assessed whether dropout looked systematic or haphazard, and auxiliary variables plausibly correlated with dropout, specifically T1 self-reported study hours and T1 negative affect, the two variables on which attriters differed from completers, were included in the FIML estimation using the saturated-correlates approach (Graham, 2003), in which each auxiliary is freely correlated with every other auxiliary, with all observed model variables, and with the residuals of the endogenous variables, but is assigned no structural path, so that it informs estimation of the missing-data mechanism without altering the hypothesized model. MAR concerns the unobserved values and is not empirically testable; adding these auxiliaries makes the assumption more defensible without verifying it. Eligibility required current undergraduate enrollment, smartphone ownership, and the ability to complete a Chinese-language self-report survey. The protocol was reviewed and approved before recruitment by the Ethics Committee of the Changsha Education Science Research Institute (approval no. CESRI-2023-012), the body with jurisdiction over non-interventional survey research in educational settings in this region; the relationship between this committee and the study site is set out in the Ethics Statement at the end of the article; informed consent at T1 covered participation across all four waves with an explicit right to withdraw at any point without penalty, and responses were linked across waves using a self-generated anonymous code rather than any personally identifying information.

### 3.3 Measures

PSU was assessed at every wave with the Smartphone Addiction Scale–Short Version (SAS-SV; Kwon et al., 2013), a 10-item measure (e.g., “Missing planned work due to smartphone use”) rated on a 6-point scale (1 = *strongly disagree* to 6 = *strongly agree*), scored as a mean composite with higher scores indicating more problematic use. SAS-SV was selected over longer PSU instruments for its brevity in repeated administration and its status as the field’s dominant measure (Harris et al., 2020).

AP was assessed at every wave with the short-form AP Scale (Yockey, 2016), a 5-item measure (e.g., “I delay the start of tasks that I do not enjoy”) rated on a 5-point scale (1 = *strongly disagree* to 5 = *strongly agree*), scored as a mean composite with higher scores indicating greater procrastination. This instrument was chosen over longer alternatives (e.g., the PASS) to minimize cumulative respondent burden across four waves; the tradeoff, less item-level redundancy to survive scalar invariance testing, is addressed in the analytic plan below.

Covariates, all measured once at T1 and regressed on both random intercepts, were gender (male/female), only-child status (yes/no; relevant given the one-child-policy-era composition of the cohort and its documented links to self-regulation and parenting-style differences), grade/year of study (1st–4th year), self-reported average weekly self-study hours (continuous, a workload/engagement proxy), trait self-control (13-item Brief Self-Control Scale, BSCS; Tangney et al., 2004; 5-point scale), and trait negative affect (Depression and Anxiety subscales of the 21-item Depression Anxiety Stress Scales, DASS-21; Antony et al., 1998; 14 items, 4-point scale). The last two operationalize the shared-dispositional-vulnerability logic behind H3 as measured covariates and rule out negative affect as an obvious third-variable confound of the trait-level PSU–AP association.

### 3.4 Common method variance

Because both focal constructs were self-reported at every wave, common method variance (CMV) was treated as a primary methodological concern. The principal diagnostic compared model fit with and without an unmeasured latent method factor loading on all indicators; a fit decrement would indicate that shared method variance, not the hypothesized structure, was driving the observed associations. We also reported Harman’s single-factor test for continuity with prior literature. However, it is a weak diagnostic on its own and was not treated as sufficient evidence of minimal CMV in isolation.

### 3.5 Analytic plan

Analyses proceeded in seven steps. First, descriptive statistics (means, SDs, skewness, kurtosis), internal consistency (Cronbach’s α, McDonald’s ω) per wave, and the full zero-order correlation matrix were computed, alongside Little’s MCAR test and the completer-versus-attriter comparison described above. Second, longitudinal measurement invariance (configural, metric, scalar) was tested separately for PSU and AP across the four waves via confirmatory factor analysis, before fitting any structural model; a partial-invariance fallback (freeing the least-invariant loading or intercept) was pre-specified if full scalar invariance was not supported, a live risk given the brevity of the AP scale.

Third, the structural model was a RI-CLPM: random intercept factors for PSU and AP (RI-PSU, RI-AP), each with unit loadings across all four wave indicators; within-person components at each wave regressed on within-person components at the immediately prior wave, separately autoregressive (α1–α3 for PSU, β1–β3 for AP) and cross-lagged (γ1–γ3 for PSU → AP, δ1–δ3 for AP → PSU); a within-person residual correlation at T1 and freely estimated residual covariances at T2–T4; RI-PSU correlated with RI-AP net of the trait self-control and trait negative affect covariates; and all six covariates regressed on both random intercepts. Consistent with standard RI-CLPM identification, each random intercept was constrained to be uncorrelated with every within-person component at every wave, so all trait-like, time-invariant variance is captured by the random intercepts, and only wave-specific deviations enter the autoregressive and cross-lagged structure. Fourth, we treated wave-level composite (mean) scores for PSU and AP as continuous manifest indicators, and estimated the model by maximum likelihood with robust (sandwich) standard errors and full-information maximum likelihood for incomplete cases, in Python 3.11 using semopy 2.3.11 (Igolkina and Meshcheryakov, 2020). We adopted a composite-level rather than fully latent RI-CLPM for reasons of identification and estimation stability, stated here rather than left implicit. A latent RI-CLPM across four waves and 15 focal items requires 60 indicators, two random intercepts, eight within-person latent factors, and a full set of longitudinal residual covariances, which this sample does not support with acceptable convergence behavior; the composite-level common-method-factor check reported in Section 4.4, which failed to identify the random-intercept correlation once only eight further loadings were added, shows concretely how quickly identification degrades at this N. The cost of the choice is real. Composite indicators carry measurement error into the structural model uncorrected; they do not inherit the invariance constraints tested in Section 4.2, and where item intercepts shift across waves, as they do for PSU, the composite’s effective origin can shift with them, which can attenuate or distort the within-person deviation scores on which every cross-lagged path is estimated. Sensitivity evidence bearing on this is reported in Section 4.2, and its limits are stated in Section 5.4. Robust standard errors are Huber-White sandwich estimates, computed as A^−1^BA^−1^ with A the observed information at the solution and B the outer product of the case-level score contributions; the model chi-square is twice the difference between the saturated full-information log-likelihood, obtained by expectation maximization on the unstructured mean vector and covariance matrix, and the log-likelihood of the fitted model; and every constrained-model comparison in Tables 1, 2 is a likelihood-ratio test on that same metric, twice the difference in log-likelihood between the constrained and the freely estimated model. Standard errors and confidence intervals for the standardized coefficients are obtained using the delta method, which differentiates each standardized quantity with respect to the full free-parameter vector and combines that gradient with the robust covariance matrix, so the uncertainty in the variance parameters that enter the standardization is carried through rather than ignored. Standardized coefficients are reported throughout for interpretability; unstandardized estimates with robust standard errors and confidence intervals for every freed parameter are provided in the Supplementary Materials, and because standardization rescales each structural path by a fixed factor, the z and *p* values are identical on the two metrics.

**Table 1:** Stationarity tests for the wave-specific Cross-Lagged paths.

Test	Δχ^2^ (df)	*p*	Conclusion
γ1 = γ2 = γ3 (δ freely estimated)	11.16 (2)	0.004	Stationarity rejected
δ1 = δ2 = δ3 (γ freely estimated)	4.85 (2)	0.088	Stationarity not rejected
Joint: γ pooled and δ pooled simultaneously	11.92 (4)	0.018	Stationarity rejected

All tests compare a constrained model against the freely estimated full model (χ^2^(45) = 67.72; see Table 5, note) using the same saturated/baseline comparison underlying every fit index in this study. The γ-equality constraint is rejected: the three wave-specific γ paths (Table 5) are not interchangeable, and γ_avg should be read as a summary of a heterogeneous set of paths rather than a single well-estimated effect. The δ-equality constraint is not rejected, so δ_avg is a defensible summary of the three δ paths.

**Table 2:** RQ1 asymmetry tests: pooled and wave-matched comparisons.

Comparison	Difference	SE	z	*p*
γ_avg − δ_avg (pooled estimates)	0.226	0.100	2.26	0.024
γ3 − δ3 (third-wave estimates only)	0.297	0.113	2.63	0.008

The pooled comparison uses γ_avg and δ_avg as reported in Table 5 (each from the model that leaves the other parameter freely estimated per Table 1); because γ_avg summarizes a heterogeneous, non-stationary set of paths, this comparison should be read as a defensibly pooled null δ set against a γ figure dominated by one wave, not two equally well-behaved single effects. The wave-matched comparison instead contrasts the two third-interval paths directly (γ3, which was individually significant, and δ3, which was not; Table 5), avoiding the pooling issue entirely at the cost of using a single interval’s estimate on each side; both comparisons point in the same direction. Both z-based comparisons treat the two paths being compared as independent. An equality-constraint likelihood-ratio test within the fitted RI-CLPM, which incorporates the full parameter covariance matrix, confirmed the wave-matched asymmetry, Δχ^2^(1) = 9.16, *p* = 0.002.

Fifth, model fit was evaluated using χ^2^, df, CFI, TLI, RMSEA (90% CI), and SRMR, against conventional benchmarks (CFI/TLI ≥ 0.95, RMSEA ≤ 0.06, SRMR ≤ 0.08). Sixth, hypotheses were tested as follows: H1 and H2 via the sign and significance of the wave-specific γ and δ paths, with pooled wave-averaged estimates (γ_avg, δ_avg) reported only after a nested chi-square stationarity test confirmed the three wave-specific paths could be defensibly constrained equal; H3 via the RI-PSU–RI-AP correlation net of the trait self-control and trait negative affect covariates; and RQ1 via a difference test between the pooled γ_avg and δ_avg estimates from a covariate-adjusted model constraining both sets of paths equal, supplemented by a wave-matched comparison of the two third-interval estimates. Both z-based difference tests treat the two estimates as independent; because they come from a single fitted model, their sampling covariance is not zero, so an equality-constraint likelihood-ratio test within the fitted RI-CLPM is also reported as the formal within-model comparison.

Seventh, four robustness checks were conducted: a comparison against a traditional CLPM without random intercepts (or the six covariates), following the comparative approach used by Burns et al. (2019) and Qi et al. (2024); a supplementary, exploratory multi-group comparison of structural paths across gender; the unmeasured-method-factor CMV check described in Section 3.4; and a re-estimation of the pooled model with a method factor added to all eight wave-level composite indicators, to test whether the focal structural paths were robust to CMV.

Finally, hypotheses and analytic plan were specified *a priori* on theoretical grounds but were not pre-registered on a public registry (e.g., OSF) prior to data collection, a limitation rather than a confirmatory, pre-registered design.

## 4 Results

### 4.1 Descriptive statistics and reliability

Retained sample size was 512 at T1, declining to 460 (T2), 436 (T3), and 422 (T4), a cumulative retention of 82.4%, consistent with the anticipated attrition band though front-loaded rather than even across intervals (10.2% dropout T1 → T2, tapering to 5.2 and 3.2%). Table 3 reports means, SDs, skewness, and per-wave reliability. PSU scores were mildly right-skewed at every wave (skewness 0.33–0.47); AP scores showed a similar, smaller skew (0.08–0.16). Internal consistency was good throughout (PSU α = 0.895–0.908; AP α = 0.918–0.931), with McDonald’s ω closely matching α at every wave. Zero-order correlations appear in the online supplementary correlation matrix; PSU and AP correlated 0.15–0.30 across all wave pairings, and both constructs correlated with trait self-control (r = −0.10 to −0.17) and trait negative affect (r = 0.03 to 0.22) in the expected directions. Attrition was strictly monotone: no participant who missed a wave returned at a later one, so the observed data reduce to four response patterns: complete at all four waves (*n* = 422), T1–T3 only (*n* = 14), T1–T2 only (*n* = 24), and T1 only (*n* = 52). Every enrolled participant therefore contributed at least the T1 wave and was retained under FIML.

**Table 3:** Descriptive statistics and internal consistency for PSU and AP by wave.

Wave	*n*	M	SD	Skewness	Kurtosis	α	ω
PSU							
T1	512	3.00	0.96	0.33	−0.48	0.895	0.896
T2	460	2.94	0.96	0.33	−0.41	0.895	0.895
T3	436	2.87	0.95	0.47	−0.34	0.899	0.899
T4	422	2.92	0.99	0.45	−0.29	0.908	0.908
AP							
T1	512	2.87	1.34	0.16	−1.29	0.927	0.927
T2	460	2.88	1.35	0.11	−1.32	0.931	0.931
T3	436	2.99	1.33	0.08	−1.28	0.918	0.918
T4	422	2.86	1.34	0.16	−1.30	0.926	0.926

PSU = Problematic Smartphone Use (SAS-SV composite, 1–6 scale); AP = Academic Procrastination (Yockey short-form composite, 1–5 scale). α = Cronbach’s alpha; ω = McDonald’s omega.

Little’s MCAR test did not reject the null of missingness completely at random, χ^2^(12) = 9.20, *p* = 0.686. A completer-versus-attriter comparison (Welch’s *t*-tests), however, found that participants who dropped out by T4 (*n* = 90) reported significantly lower T1 study hours (*t*(130.65) = 2.26, *p* = 0.025) and significantly higher T1 negative affect (*t*(129.48) = −2.88, *p* = 0.005) than completers; gender, only-child status, grade, self-control, and T1 PSU/AP scores did not differ between groups. Because these two variables predict dropout, missingness is not completely at random despite the non-significant Little’s test, and we entered both as auxiliary variables in the FIML estimation by the saturated-correlates method described in Section 3.2. This makes MAR conditional on the observed data a more defensible working assumption than MCAR, but MAR itself concerns the unobserved values and cannot be confirmed empirically; the possibility that dropout depends on unmeasured factors remains open and is listed among the study’s limitations.

### 4.2 Longitudinal measurement invariance

Table 4 reports configural, metric, and scalar invariance fit for PSU and AP. Metric invariance was supported for both constructs (ΔCFI ≤ 0.003). Scalar invariance was supported for AP (ΔCFI = 0.008) but not for PSU (ΔCFI = 0.018); freeing the least-invariant PSU item’s intercept (partial scalar) did not resolve this (ΔCFI = 0.015 relative to metric), so PSU’s final retained invariance level is metric only, the reverse of the *a priori* risk flagged in the Method section. Because the structural RI-CLPM is estimated on wave-level composite scores rather than the fully invariance-constrained multi-indicator model, this does not block the structural analysis, but it does bear on how the PSU results should be read, and the implication is spelled out here rather than deferred to a limitations sentence. Scalar non-invariance means the expected item response at a given latent PSU level shifts across waves; a composite computed with fixed unit weights then has an origin that shifts with it, so a wave-to-wave change in a participant’s PSU composite blends genuine within-person change with a measurement-side shift. Because the RI-CLPM estimates its cross-lagged paths on exactly those within-person deviations, the PSU → AP estimates, the significant T3 → T4 path included, are less secure than they would be under full scalar invariance, and mean-level comparisons of PSU across waves at the latent-intercept level are not licensed at all. Two considerations bound how consequential this is. First, metric invariance did hold, so the loadings, and with them the relative weighting of items within the composite, are stable across waves; it is the intercepts, not the structure, that move. Second, the flagged item contributes very little unique variance to the composite: a nine-item PSU composite omitting it correlates 0.994 or above with the retained ten-item composite at every wave (r = 0.994, 0.994, 0.994, and 0.995 at T1 through T4), so a respecification that dropped the item would leave the within-person deviation scores, and hence the structural estimates, essentially unchanged. Because a near-identical composite is not the same thing as a near-identical set of structural estimates, the RI-CLPM was also refitted on the nine-item composite directly; that refit is reported in Section 4.4 and leaves the focal estimates essentially unchanged. Neither the composite correlation nor the refit substitutes for a fully latent RI-CLPM carrying partial-invariance constraints into the structural model, which this sample size did not support (Section 3.5), and the PSU-side longitudinal interpretation is qualified accordingly throughout; Section 5.4 states the residual risk.

**Table 4:** Longitudinal measurement invariance tests for PSU and AP.

Model	χ^2^	df	CFI	TLI	RMSEA	SRMR	ΔCFI	ΔRMSEA
PSU								
Configural	715.12	674	0.996	0.995	0.011	0.028	—	—
Metric	767.30	701	0.993	0.992	0.014	0.033	0.003	0.003
Scalar	971.01	731	0.975	0.973	0.025	0.033	0.018	0.012
Partial scalar	940.88	728	0.978	0.976	0.024	0.033	0.015	0.010
AP								
Configural	178.40	134	0.995	0.993	0.025	0.016	—	—
Metric	186.85	146	0.996	0.994	0.023	0.018	−0.0004	−0.002
Scalar	271.84	161	0.988	0.986	0.037	0.017	0.008	0.013

ΔCFI/ΔRMSEA computed relative to the immediately less-constrained model (metric relative to configural; scalar/partial scalar relative to metric). Full scalar invariance was supported for AP (ΔCFI ≤ 0.01) but not for PSU; the partial-scalar fallback (freeing item 7’s intercept) also did not bring PSU within the ΔCFI ≤ 0.01 criterion, so PSU’s retained invariance level is metric only.

### 4.3 RI-CLPM structural results

The full RI-CLPM (six covariates regressed on both random intercepts) fit the data adequately, χ^2^(45) = 67.72, *p* = 0.016, CFI = 0.994, TLI = 0.988, RMSEA = 0.031, 90% CI [0.014, 0.046], SRMR = 0.022. Standardized structural paths appear in Table 5 and Figure 2.

**Table 5:** Standardized RI-CLPM structural path estimates (full model with covariates).

Path	β	SE	z	p	95% CI
Autoregressive: PSU (α)					
wPSU1 → wPSU2	−0.061	0.164	−0.37	0.711	[−0.38, 0.26]
wPSU2 → wPSU3	0.273	0.098	2.79	0.005	[0.08, 0.46]
wPSU3 → wPSU4	0.277	0.074	3.77	<0.001	[0.13, 0.42]
Autoregressive: AP (β)					
wAP1 → wAP2	0.114	0.137	0.83	0.406	[−0.16, 0.38]
wAP2 → wAP3	0.158	0.111	1.42	0.157	[−0.06, 0.38]
wAP3 → wAP4	0.228	0.108	2.11	0.034	[0.02, 0.44]
Cross-lagged: PSU → AP (γ, H1)					
wPSU1 → wAP2 (γ1)	0.097	0.111	0.87	0.384	[−0.12, 0.31]
wPSU2 → wAP3 (γ2)	−0.013	0.113	−0.12	0.906	[−0.24, 0.21]
wPSU3 → wAP4 (γ3)	0.374	0.080	4.68	<0.001	[0.22, 0.53]
Pooled γ_avg†	0.213	0.066	3.22	0.001	[0.08, 0.34]
Cross-lagged: AP → PSU (δ, H2)					
wAP1 → wPSU2 (δ1)	−0.259	0.168	−1.54	0.123	[−0.59, 0.07]
wAP2 → wPSU3 (δ2)	0.126	0.101	1.24	0.213	[−0.07, 0.32]
wAP3 → wPSU4 (δ3)	0.077	0.079	0.97	0.331	[−0.08, 0.23]
Pooled δ_avg†	−0.013	0.075	−0.17	0.863	[−0.16, 0.13]
Residual covariances (within-person)					
wPSU1 ↔ wAP1	−0.197	0.139	−1.42	0.157	[−0.47, 0.08]
wPSU2 ↔ wAP2	−0.338	0.137	−2.47	0.014	[−0.61, −0.07]
wPSU3 ↔ wAP3	0.145	0.081	1.79	0.073	[−0.01, 0.30]
wPSU4 ↔ wAP4	0.181	0.070	2.57	0.010	[0.04, 0.32]
Random intercept correlation (H3)					
RI-PSU ↔ RI-AP (zero-order)	0.277	0.048	5.74	<0.001	[0.18, 0.37]
RI-PSU ↔ RI-AP (net of self-control, negative affect)	0.250	0.049	5.08	<0.001	[0.15, 0.35]

β is the standardized coefficient throughout, and all 95% CIs are β ± 1.96 × SE on the standardized metric shown in this table. SE is computed for every row by the delta method. Each standardized quantity is differentiated with respect to the full free-parameter vector, and that gradient is combined with the robust sandwich covariance matrix. This propagates the uncertainty in the variance parameters that enter the standardization, which a linear rescaling of the unstandardized SE does not; the two agree closely for the autoregressive and cross-lagged rows and differ more for the pooled rows. z and *p* follow from β/SE. Unstandardized estimates with robust standard errors and confidence intervals for every freed parameter are given in the Supplementary Materials. † Pooled γ_avg is from a model constraining only the three γ paths equal (δ left freely estimated); pooled δ_avg is from the mirror-image model constraining only the three δ paths equal (γ left freely estimated). Each is reported from the model that does not also constrain the other parameter, since Table 1 shows the γ-equality constraint is not statistically justified and imposing it needlessly would distort the other pooled estimate. Estimates are from the full RI-CLPM including all six T1 covariates (gender, only-child status, grade, study hours, self-control, negative affect) regressed on both random intercepts; covariate-to-random-intercept path estimates are reported in Table 6. Model fit: χ^2^(45) = 67.72, *p* = 0.016, CFI = 0.994, TLI = 0.988, RMSEA = 0.031, 90% CI [0.014, 0.046], SRMR = 0.022.

**Figure 2:**
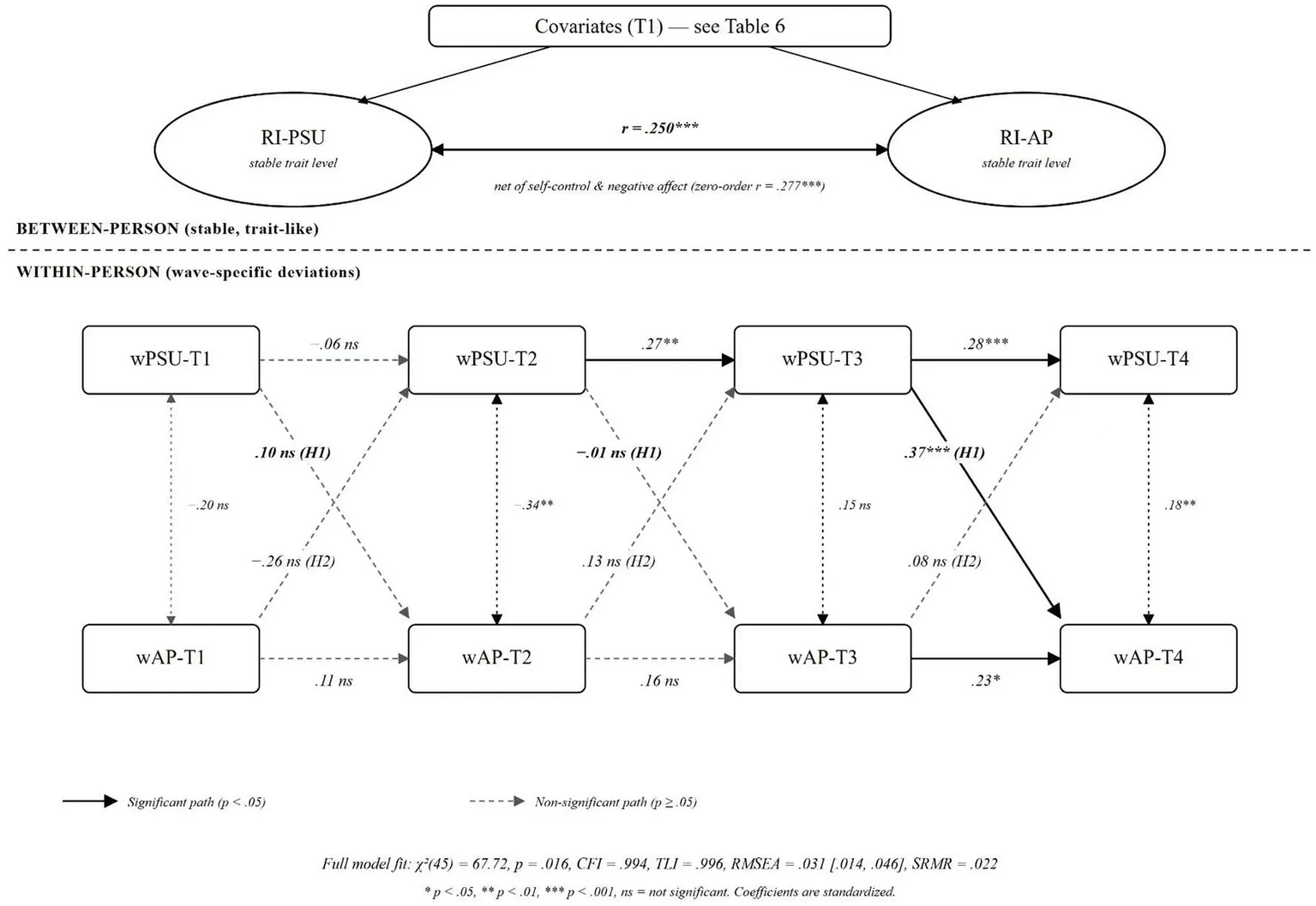
Standardized RI-CLPM structural results. Solid arrows denote *p* < 0.05; dashed arrows denote non-significant paths. Coefficients are standardized; full model fit statistics appear beneath the figure.

Two of the four within-person residual covariances warrant a note before the cross-lagged paths: T1 and T2 were negative (T2 significantly so: wPSU2 ↔ wAP2, β = −0.338, *p* = 0.014), while T3 and T4 were positive, with only the T4 estimate reaching significance (β = 0.181, *p* = 0.010) and the T3 estimate of the same sign and similar size but short of it (β = 0.145, *p* = 0.073). This sign reversal is not predicted by the present model, is reported descriptively here, and is given a substantive reading in Section 5.4, where it is treated as a live alternative explanation for the interval-specific cross-lagged result rather than as an incidental anomaly.

Stationarity across waves was tested directly rather than assumed (Table 1). Constraining γ1 = γ2 = γ3 rejected stationarity for the γ paths, Δχ^2^(2) = 11.16, *p* = 0.004; the mirror-image test on δ did not, Δχ^2^(2) = 4.85, *p* = 0.088. Pooling is accordingly not statistically defensible for γ, and pooled γ_avg below is reported only as a rough summary of a heterogeneous set of paths; pooling is defensible for δ, reported as a genuine single estimate. γ_avg and δ_avg are each taken from the model pooling only that one parameter, leaving the other freely estimated, rather than from a single model pooling both simultaneously (Table 5, note).

#### 4.3.1 H1 (PSU → AP)

The verdict depends on which wave is asked about, not on a single pooled number. Only the third wave-specific path (wPSU3 → wAP4) was individually significant, β = 0.374, *p* < 0.001, 95% CI [0.217, 0.531]; the first two were not, β = 0.097, *p* = 0.384, and β = −0.013, *p* = 0.906, and the stationarity test above confirms these three paths are not interchangeable estimates of one effect. The pooled summary, γ_avg = 0.213, SE = 0.066, *p* = 0.001, is positive and significant, but because the equality constraint underlying it has been rejected, it is reported as a descriptive average over three heterogeneous paths and is not treated as the evidence for H1; the wave-specific estimates are that evidence. H1 accordingly receives partial support, confined to the final T3 → T4 interval, and no claim is made that within-person increases in PSU predict later AP generally across the academic year.

#### 4.3.2 H2 (AP → PSU)

None of the three wave-specific δ paths reached significance: β = −0.259, *p* = 0.123 (T1 → T2, opposite in sign to the hypothesized direction); β = 0.126, *p* = 0.213 (T2 → T3); β = 0.077, *p* = 0.331 (T3 → T4). Unlike γ, pooling is statistically defensible here (stationarity test above). The resulting single estimate is essentially null, δ_avg = −0.013, SE = 0.075, *p* = 0.863, smaller and opposite in sign from a naive joint-pooling specification (δ_avg = 0.047 if γ is forced equal at the same time, an estimate contaminated by that already-rejected constraint; Table 5, note). H2 is not supported. Given the design-stage power estimate of 0.81 for an effect of the size hypothesized (Section 4.4), this result should be read as a reasonably informative failure to detect the AP → PSU pathway at a three-month lag in this sample, rather than as evidence that the pathway does not exist.

#### 4.3.3 H3 (trait-level RI-PSU–RI-AP correlation)

The random intercepts were significantly correlated both without covariates, r = 0.277, SE = 0.048, *p* < 0.001, 95% CI [0.182, 0.371], and in the full model, in which all six T1 covariates (gender, only-child status, grade, study hours, trait self-control, trait negative affect) are regressed on both random intercepts, r = 0.250, SE = 0.049, *p* < 0.001, 95% CI [0.153, 0.346], only modestly attenuated. Throughout this article, the adjusted random-intercept correlation refers to the six-covariate model; trait self-control and trait negative affect are named in the wording of H3 because they operationalize the shared-vulnerability account, not because the adjustment is restricted to them. Self-control significantly predicted both random intercepts (RI-PSU β = −0.122, *p* = 0.018; RI-AP β = −0.132, *p* = 0.003), negative affect significantly predicted RI-PSU (β = 0.195, *p* < 0.001) but not RI-AP (β = 0.026, *p* = 0.570), and study hours significantly predicted RI-AP (β = −0.098, *p* = 0.032) but not RI-PSU (β = −0.070, *p* = 0.128); full covariate estimates appear in Table 6. H3 is supported. RI-PSU and RI-AP variances were 0.656 (SE = 0.044, *p* < 0.001) and 1.527 (SE = 0.069, *p* < 0.001); relative to model-implied total variance at T1, the random intercepts account for an estimated 74.6% (PSU) and 87.4% (AP). Most of what these composites measure at any single wave is the stable, trait-like component.

**Table 6:** Standardized covariate effects on the random intercepts.

Covariate	RI-PSU β	RI-PSU p	RI-AP β	RI-AP p
Gender	0.072	0.109	0.066	0.142
Only-child status	−0.005	0.917	0.033	0.461
Grade	−0.028	0.548	0.033	0.458
Study hours	−0.070	0.128	−0.098	0.032
Self-control	−0.122	0.018	−0.132	0.003
Negative affect	0.195	<0.001	0.026	0.570

#### 4.3.4 RQ1 (γ vs. δ asymmetry)

A difference test of the pooled γ and δ estimates returned a γ estimate larger than δ, difference = 0.226, SE = 0.100, z = 2.26, *p* = 0.024 (Table 2). Because pooled γ summarizes a heterogeneous, non-stationary set of paths, this is more precisely a comparison between a defensibly pooled null δ and a γ figure dominated by one wave; a wave-matched comparison using only the two third-interval estimates (γ3, which was significant, and δ3, which was not) avoids that asymmetry and reaches the same conclusion, difference = 0.297, SE = 0.113, z = 2.63, *p* = 0.008 (Table 2). Both comparisons point the same way; the PSU → AP estimate exceeds the AP → PSU estimate in each case. An equality-constraint likelihood-ratio test within the RI-CLPM confirmed the wave-matched asymmetry: constraining γ3 = δ3 degraded fit significantly, Δχ^2^(1) = 9.16, *p* = 0.002. This model-based test incorporates the full parameter covariance matrix and does not require the independence assumption embedded in the z comparisons. The γ side of all three comparisons rests on a single interval, so the asymmetry is established for the T3 → T4 window rather than as a general property of the two pathways across the year.

### 4.4 Robustness and supplementary checks

A traditional CLPM without random intercepts fit these data poorly, χ^2^(12) = 273.25, CFI = 0.933, TLI = 0.843, RMSEA = 0.206, and produced substantially inflated autoregressive paths relative to the RI-CLPM for both PSU (66–92% shrinkage once random intercepts were added) and AP (74–87% shrinkage), alongside small but “significant” cross-lagged paths in both directions at nearly every wave. This comparison model excluded the six covariates present in the RI-CLPM. A supplementary CLPM retaining all six covariates produced the same pattern: autoregressive paths exceeded 0.77 throughout, and five of the six cross-lagged paths were significant, the exception being the first-interval AP → PSU path (β = 0.011, *p* = 0.709), which was the one non-significant cross-lagged path in the model without covariates as well. The inflation relative to the RI-CLPM is therefore attributable to the omission of random intercepts rather than covariate specification differences: absent the random-intercept decomposition, the data would appear to show pervasive, symmetric bidirectional reciprocity that the RI-CLPM’s within-person estimates do not support.

Two CMV checks were run at different levels of the data. At the item level, an unmeasured latent method factor added to a T1-only CFA of all 15 items improved fit over a model without one, Δχ^2^(15) = 31.39, *p* = 0.008, accounting for an average of 4.8% of item variance (range 0.0–25.5%), a detectable but modest common-method component; Harman’s single-factor test on the same items found one factor accounted for 33.2% of variance, below the conventional 50% flag threshold. At the composite level, a method factor added to all eight wave-level composite indicators of the joint-pooled structural model improved fit, Δχ^2^(8) = 22.34, *p* = 0.004, so a common-method component is present rather than absent. That specification is only weakly identified, however, and the pooled cross-lagged estimates do not survive it in recognizable form: γ_avg falls from 0.205 to 0.101 while δ_avg rises from 0.047 to 0.459, and the method-factor loadings return standard errors between 0.13 and 3.34 times their point estimates. With only eight composite indicators, this check cannot establish whether either set of estimates is inflated by shared method variance, so we report it as inconclusive rather than as evidence in either direction. A Monte Carlo power simulation (200 replications, all of which converged), using the fixed population parameters specified at the data-generation stage, estimated power above 0.99 to detect the pooled H1 effect and 0.81 to detect the pooled H2 effect; this design was well powered for H1 and adequately, if less comfortably, powered for an effect the size of H2’s. An exploratory gender comparison found the focal paths statistically equivalent across men (*n* = 265) and women (*n* = 247) with one exception: the wPSU3 ← wAP2 path differed significantly by gender (*z* = 2.36, *p* = 0.019), positive and significant for women (β = 0.254, *p* < 0.001) but non-significant for men (β = −0.148, *p* = 0.374). One further female-subsample path (wPSU2 ← wAP1) returned an implausibly large standard error (*SE* = 3.47, an order of magnitude above every other path), consistent with a boundary or near-unidentified estimate rather than a genuine effect, and is not interpreted.

Two further checks address the measurement and clustering concerns directly. First, because a composite that correlates almost perfectly with another need not produce the same structural estimates, the full RI-CLPM was refitted with PSU scored from the nine items that survive the partial-scalar model, dropping the item whose intercept was freed. Fit was equivalent, χ^2^(45) = 66.51, CFI = 0.995, RMSEA = 0.031, and the focal estimates were essentially unchanged: the T3 → T4 γ path was β = 0.378, *p* < 0.001 against 0.374 in the ten-item model, no other cross-lagged path changed by more than 0.02, the adjusted random-intercept correlation was 0.253 against 0.250, and the stationarity tests reached the same conclusions (γ pooling rejected, Δχ^2^(2) = 10.80, *p* = 0.005; δ pooling not rejected, Δχ^2^(2) = 4.29, *p* = 0.117). The scalar-invariance failure therefore does not appear to be driving the structural results. However, this remains a composite-level check rather than the latent model that would test the point directly. Second, because course-section identifiers were not retained, the consequences of the unmodelled clustering were bounded arithmetically rather than corrected: with 14 sections and a mean cluster size of about 37, a design effect of 1 + (m − 1)ρ inflates the standard error of the T3 → T4 γ path from 0.080 to 0.133 at ρ = 0.05 (*p* = 0.005) and to 0.171 at ρ = 0.10 (*p* = 0.029); the path loses significance at ρ ≈ 0.13, a design effect of about 5.7. An intraclass correlation that large would be unusual for course sections, so the central estimate is unlikely to rest on clustering alone, but this calculation assumes one common design effect and is not a substitute for a cluster-robust correction.

## 5 Discussion

This study asked whether PSU and AP genuinely precede each other within individual Chinese undergraduates across a full academic year, once each person’s stable trait-like standing is statistically separated from wave-to-wave fluctuation. The answer is interval-specific, and asymmetric only within the interval that produced it: PSU predicted later within-person increases in AP in the final wave interval and in neither of the two before it; AP did not predict later within-person increases in PSU at any interval; and the well-documented cross-sectional PSU–AP association turned out to be substantially, though not entirely, a stable between-person phenomenon. A traditional cross-lagged panel model fit to the same data would have suggested pervasive, significant, bidirectional reciprocity at nearly every wave; the within-person decomposition tells a more selective story.

### 5.1 Hypothesis-by-hypothesis interpretation

H1 predicted that within-person increases in PSU would positively predict later within-person increases in AP. The result is wave-specific rather than uniform: only the final-wave path was individually reliable (Section 4.3), a difference the stationarity test confirms is real rather than sampling noise. One interpretation, consistent with I-PACE’s account of a use pattern that compounds over time, holds that a full academic year’s accumulated engagement is needed before an executive-control cost becomes detectable in the procrastination composite. That interpretation is plausible but not established here; a single wave carrying most of the pattern is equally consistent with a genuinely later-emerging process, a chance fluctuation, or an unmodeled event specific to that term’s final weeks (see the residual-covariance discussion below). H1 receives partial support, confined to the final wave interval. Because the one significant interval is also the one that closes the academic year, and because the within-person residual covariance between the two constructs changes sign at the same semester boundary (Section 5.4), the interval-specific reading is preferred here over any claim that PSU predicts later AP across the year as a whole.

H2 predicted the reverse: within-person increases in AP predicting later within-person increases in PSU. None of the three wave-specific paths reached significance, and the pooled null estimate (δ_avg = −0.013, *p* = 0.863) is a defensible single quantity rather than a rough summary. This narrows the claim in Hong et al. (2021), whose younger Chinese adolescent sample, living under different autonomy and monitoring conditions, showed AP preceding PSU in a mediation model that did not separate trait from state variance; because the analytic and population differences changed at once, this study cannot credit the non-replication to either alone. The Monte Carlo power check indicated about 81% power to detect an effect the size originally hypothesized, so the failure to detect one is a reasonably informative null. However, an effect appreciably smaller than the one hypothesized could still have gone undetected.

H3 predicted that the random-intercept correlation between PSU and AP would remain significant after controlling for trait self-control and trait negative affect. It did (net-of-covariates *r* = 0.250, *p* < 0.001), attenuated only modestly from the unconditional correlation (*r* = 0.277). Trait self-control independently predicted both random intercepts in the expected direction, whereas trait negative affect predicted the PSU random intercept (β = 0.195, *p* < 0.001) but not the AP random intercept (β = 0.026, *p* = 0.570). Hence, the dispositional-vulnerability account holds up only partly and asymmetrically. These two measured traits absorb only a fraction of the shared trait-level variance, leaving a sizable residual association that this study cannot further decompose.

RQ1 asked whether the pooled γ and δ estimates differ in magnitude. Given the stationarity results above, this is better understood as a defensibly pooled null δ set against a γ summary dominated by one wave rather than two equally well-behaved single effects. A wave-matched comparison using only the third-wave estimates, avoiding the pooling issue, reaches the same conclusion (Table 2), reassuring that the pooled result is not an artifact of how γ was summarized. Section 2.7 flagged one plausible basis for this asymmetry before the data were analyzed: I-PACE’s escalating-use logic implies a compounding capacity-erosion process, while the emotion-regulation account of procrastination implies a more episodic, acute response that may not aggregate into a detectable average effect at a three-month grain. RQ1 was specified as an open question because that reasoning was judged too thin to commit to in advance; the result is consistent with it, but consistency after the fact is weaker evidence than an advance prediction would have been. An equality-constraint likelihood-ratio test within the fitted RI-CLPM confirmed this asymmetry without requiring the independence assumption embedded in the z comparisons, Δχ^2^(1) = 9.16, *p* = 0.002. The asymmetry is nonetheless specific to the T3 → T4 window and should be read as an interval-level finding rather than a property of the two pathways over the year.

One supplementary result complicates a fully uniform reading of the null H2 finding: the second-wave AP-to-PSU path differed significantly by gender, positive and significant for women but flat for men. This exploratory result suggests the pooled, gender-collapsed null may mask heterogeneity that a larger, gender-stratified design would resolve more directly.

### 5.2 Theoretical implications

The clearest theoretical payoff here is methodological: Hamaker et al.'s (2015) critique of the standard cross-lagged panel model is demonstrated concretely in this literature rather than merely argued for in the abstract. The traditional CLPM fit these same composite scores poorly and produced large, apparently robust autoregressive and cross-lagged paths in both directions; the RI-CLPM’s within-person autoregressive paths shrank by roughly two-thirds to nine-tenths once trait-like stability was partitioned out. A field relying almost entirely on cross-sectional correlations and a single one-directional mediation study should treat this shrinkage as a caution against reading prior CLPM-style or cross-sectional evidence in this domain as settled proof of a strong, symmetric, reciprocal process.

Substantively, the asymmetric pattern is consistent with I-PACE’s capacity-erosion logic operating on a longer timescale than the emotion-regulation account of procrastination does at the three-month grain tested here. That reading carries a caution: a design with one fixed lag cannot distinguish “this pathway takes about a year to show up” from “this pathway does not exist,” and building a timescale story after seeing which wave was significant is not a genuine test of anything. The asymmetry is estimated consistently across both comparisons reported here, but it rests on a single interval’s γ estimate and is best described as an interval-specific feature of this dataset rather than an established general property of the two constructs; the timescale explanation for it is plausible but unconfirmed.

The between-person finding earns its own place in the theoretical story, independent of the timing question above. A substantial share of the widely-cited PSU–AP correlation belongs to the same stable-disposition family both frameworks already invoke, and self-control functions as a shared, measurable anchor for at least part of it. Future integrative work on PSU and academic self-regulation would do well to build dispositional vulnerability explicitly into the model, as this study did.

This study treated I-PACE and the self-regulatory/emotion-regulation account of procrastination as complementary lenses that converge on a shared self-regulatory resource rather than a single jointly derived mechanism. Hence, the results here test each hypothesis but not the framework that houses them. A single shared depletion process and two distinct processes drawing on the same broad resource could both produce the asymmetric pattern found here, so the asymmetry itself cannot adjudicate between these accounts; that would need a design measuring the resource directly.

The common-method-variance results add a further point for future theorizing. A latent method factor explained a modest but detectable share of item variance, concentrated much more heavily in a handful of PSU items than spread evenly across the scale, more consistent with a few items sharing incidental wording or response-style variance than wholesale contamination of the substantive constructs. Adding a method factor to the pooled structural model improved fit, so a common-method component is present rather than absent. It does not license the further claim that the cross-lagged findings survive that component: the composite-level method-factor specification is only weakly identified, and the pooled estimates move too far under it to be read as stable (Section 4.4). Neither the cross-lagged paths nor the trait-level correlation can therefore be described as robust to shared method variance on the evidence available here, and settling the question would need multiple indicators per construct at each wave, or a use measure that is not self-reported.

### 5.3 Practical implications

The evidentiary basis for any practical recommendation here is modest: one reliable wave-specific path for H1, no reliable path for H2, and an attenuated but still-significant trait correlation. With that ceiling acknowledged, the pattern still points somewhere. On the evidence here, and only within the single interval in which an effect was detected, interventions aimed at reducing problematic phone use (app-limiting tools, structured phone-free study blocks, digital literacy programming) have a marginally more direct empirical foothold in breaking a PSU-to-AP spillover than interventions aimed narrowly at procrastination, and the calendar-linked explanations set out in Section 5.4 would account for the same pattern without any spillover at all. That foothold consists of one significant cross-lagged path in one of three intervals, in one cohort at one institution, in a design that cannot support causal inference; it does not establish an intervention sequence. The between-person finding argues against a phone-use-only strategy: because a meaningful share of the PSU–AP association sits in the stable, between-person component and is linked there to trait self-control, programs building general self-regulatory capacity (implementation-intention training, structured goal-setting, brief self-control interventions) plausibly address the chronic co-occurrence of both behaviors in a way a phone-specific intervention alone would not. None of the licenses a causal claim from a correlational design; it is a plausibility argument for where a future intervention trial would have the best prior odds, and the intervention families named are illustrative candidates rather than an evidence-backed program. Everything in this subsection should be read as a set of hypotheses for future intervention trials to test, not as guidance for university policy.

Timing follows from the wave-specific pattern, though this is a hypothesis for future intervention-timing research to test rather than a settled recommendation, since it could reflect something particular to this cohort’s calendar: because the significant PSU-to-AP path emerged only in the spring-semester interval, a phone-use intervention delivered early in the fall, before any detectable spillover accumulates, may have more room to prevent the pathway from developing than one delivered after problems are already visible in the spring.

### 5.4 Limitations

Measurement limitations come first. PSU did not achieve scalar measurement invariance across waves, and the partial-invariance fallback did not resolve this; only metric invariance was supported. The structural RI-CLPM was estimated on composite scores rather than the fully invariance-constrained latent model, which does not automatically invalidate the cross-lagged and random-intercept estimates, but if PSU’s item intercepts genuinely shift across waves, the composite’s effective zero-point can shift with them, potentially biasing the within-person deviation scores. Claims about mean-level change in PSU accordingly rest on weaker ground than for AP, which achieved full scalar invariance. Both constructs were measured exclusively via self-report, and self-reported smartphone use in particular correlates only modestly with logged, objective usage data so that some PSU-related findings may reflect self-perception more than device behavior. Common method variance compounds this, and here the manuscript has to state a negative result plainly: the focal paths were not shown to be robust to it. The model carrying a method factor fit better than the model without one, so a common-method component is present rather than absent; the pooled cross-lagged estimates did not hold their values once it was added; and the specification is too weakly identified at eight composite indicators for either those paths or the trait-level correlation to be re-estimated dependably under it (Sections 4.4 and 5.2). Shared-method inflation of either therefore cannot be ruled out, and the central claim of this article should be read with that unresolved. Two further points belong here. The decision to fit the RI-CLPM on composites rather than latent variables (Section 3.5) means the structural model does not inherit the invariance testing reported in Section 4.2 at all: the measurement analysis and the structural analysis are linked by argument rather than by shared constraints. The strongest sensitivity evidence available for the PSU scalar-invariance problem is the refit of the structural model on the item-reduced composite (Section 4.4), which leaves the focal estimates essentially unchanged; a fully latent or partial-invariance RI-CLPM, which would test the problem directly, would not converge acceptably at this sample size. A replication with a larger sample, or with a longer PSU instrument affording more item-level redundancy, should fit the latent model and report the focal paths under partial-invariance constraints directly.

Design limitations come next. The sample was drawn from a single Chinese university via cluster sampling of course sections, but section identifiers were not retained in the analytic dataset. As a result, neither a cluster-robust correction nor a multilevel specification could be applied, and the data contain no proxy for a clustering variable. This matters in three specific ways, not as a generic caveat. Standard errors throughout should be treated as a plausibly optimistic lower bound, bearing most directly on the T3 → T4 γ path that carries this study’s central claim. Section 4.4 quantifies how much that matters: with 14 sections and an average of about 37 students in each, the intraclass correlation would have to reach about 0.13 before that path lost significance, which corresponds to a design effect of roughly 5.7 and an inflation of its standard error by a factor of about 2.4. That is a large intraclass correlation for course sections, so the central estimate is unlikely to be an artifact of clustering alone, but the calculation assumes a common design effect and is no substitute for the cluster-robust correction the data do not permit. Students within a section share an instructor, an assessment schedule, and a workload profile, so section membership could plausibly induce shared variation in AP in particular and, through timetable and assessment pressure, in PSU as well, and could equally shape the wave-specific estimates. And because attrition may itself cluster, whole sections dropping out through timetable changes rather than through individual disengagement, the missing-data analysis in Section 4.1 inherits the same limitation. Retaining section identifiers is a straightforward design fix and is recommended for any replication. The three-month wave spacing matched the rhythm of the Chinese academic semester rather than a validated causal window for PSU–AP spillover, and a shorter or longer lag might reveal a different pattern for either direction, particularly the undetected AP-to-PSU pathway. Statistical power for the pooled δ path was about 81% against an effect of the hypothesized size, so that null result carries more information than a bare non-detection. However, it still means “not detected at this sample size and time lag,” not a confirmed absence of an effect. The authors specified hypotheses *a priori* on theoretical grounds but did not pre-register them on a public registry, a transparency gap relative to a fully confirmatory design.

Two further points are worth flagging. The within-person residual covariance between PSU and AP was significantly negative at T2 and significantly positive at T4, with the T3 estimate positive but not significant (*p* = 0.073), a reversal not explained by the present model; the reversal deserves more than a descriptive note, because it coincides exactly with the boundary separating the two non-significant early γ paths from the significant final one. T1 and T2 sit within the Fall semester, and T3 and T4 within the Spring, and several unmeasured, calendar-linked processes could produce both patterns at once. Examination periods concentrate workload at the end of each semester, but end-of-year assessment in the Spring typically carries more consequence than its Fall counterpart. The winter vacation falls inside the T2 → T3 interval. It removes the ordinary structure of coursework, so the wave-to-wave deviations that interval is built from are not comparable to those in the two within-semester intervals. Seasonal variation in workload, in discretionary time, and in the salience of progression or graduation decisions all move with the same boundary. Any of these would generate a shared, unmodeled disturbance raising PSU and AP together in the Spring while leaving them uncorrelated or negatively related in the Fall, and would also make a PSU → AP path appear specifically in the final interval. This is a live alternative to the cumulative-timescale interpretation offered in Section 5.2, and nothing in the present design distinguishes the two, since no institutional-event, assessment-load, or term-calendar covariate was collected. A replication that records assessment dates and workload at each wave, or that staggers wave timing across cohorts so that calendar position is not confounded with elapsed time, could separate them. The gender and traditional-CLPM comparisons were exploratory and supplementary, and one gender-specific subsample path returned an implausibly large standard error, most likely a boundary or near-unidentified estimate rather than a genuine effect, and was excluded from interpretation. RI-CLPM itself remains subject to ongoing methodological debate, including concern that its between-person component can behave unexpectedly under certain misspecifications; the model here followed current best-practice recommendations but is not immune to that broader discussion.

### 5.5 Future directions

An intensive longitudinal design, daily or weekly experience sampling over a shorter window, could test whether the undetected AP-to-PSU pathway operates at a faster timescale, and whether the PSU-to-AP asymmetry holds, strengthens, or reverses at finer temporal grain. An experimental design, randomizing students to a phone-use-reduction condition, a self-regulation-training condition, both, or control, would be needed to move from temporally-ordered associations to an actual causal test, directly evaluating the intervention-timing hypothesis raised in Section 5.3.

A second pair of extensions targets measurement: replication with objective, logged smartphone-use data alongside self-report, across multiple institutions with cluster identifiers retained for a proper robustness correction, would address the common-method, self-report, and single-site concerns together; and a longer or reconstructed PSU item set designed to achieve full scalar invariance would let mean-level change in PSU eventually be assessed with the confidence already available for AP.

A final extension follows from Section 5.2: this study inferred a capacity-erosion mechanism from temporal precedence alone, without measuring executive control or momentary self-regulatory depletion directly. A design adding a repeated, brief measure of momentary self-control alongside PSU and AP at every wave could test depletion as an explicit within-person mediator instead of an inferred bridge. It might help explain why the PSU-to-AP pathway concentrated in the final wave.

## 6 Conclusion

In this study, PSU and AP are among the most reliably correlated behaviors in the student self-regulation literature. Until now, that reliability has rested almost entirely on cross-sectional snapshots and a single one-directional longitudinal study. This study took that correlation apart. Across a full academic year, Chinese undergraduates whose problematic smartphone use rose within themselves went on to show more procrastination in one interval only, the final one, with the two earlier intervals showing no such effect and a formal test confirming the three are not equivalent; the reverse pathway was not detected at this three-month grain, a non-detection that is reasonably informative given roughly eight in ten power against an effect of the hypothesized size, though short of proof of absence. Where it trended at all in the first interval, it trended opposite to what theory predicted. A meaningful share of why the two behaviors travel together at all turned out to be a stable, trait-like phenomenon tied in part to self-control, surviving, only modestly reduced, after controlling for the two most obvious dispositional confounds available.

Fit the same data to a traditional cross-lagged panel model, without separating trait from state, and the picture changes: stability paths inflate and significant cross-lagged effects in both directions at nearly every wave, overstating the case for a strong, symmetric feedback loop. The random-intercept decomposition tells a more selective story, in which one directional pathway carries the dynamic signal at one point in the year, the reverse pathway does not clear the bar this design could detect, and a sizable share of the association is simply about which students are chronically higher on both behaviors to begin with.

For a field that has built a large cross-sectional literature on this pairing but almost no longitudinal one, this study offers a first four-wave test of both directions at once, net of trait-level confounding. Its central lesson is interval-specificity and partial trait confounding rather than the fully reciprocal, self-reinforcing cycle that cross-sectional correlations alone might suggest; the directional asymmetry it also found belongs to the one interval that produced it, and the reverse pathway is undetected rather than ruled out. A single institution, a self-report-only measurement approach, unaddressed course-section clustering, and a three-month wave spacing all bound what this study can claim, and the null AP-to-PSU pathway, reasonably well powered against the hypothesized effect, still deserves a second look at a shorter lag. What remains is narrower but useful: the next study on this pairing does not need another cross-sectional correlation or single-direction mediation model, but a design, like this one, that can tell trait from state and test both directions at once, the only way this literature will find out whether the two behaviors actually feed each other over time, or have simply been keeping the same company all along.

## Data Availability

The datasets analysed for this study consist of repeated individual-level self-report measures of problematic smartphone use, academic procrastination, trait self-control, and negative affect. Participants consented at enrolment to the use of their responses for the research purposes described to them; they did not consent to unrestricted public release of their individual response records, and the approving ethics committee did not authorise open deposit of individual-level data. Fully de-identified individual-level data, the analysis scripts, and the full model syntax will therefore be made available to qualified researchers under a controlled-access arrangement, on reasonable request to the corresponding author and subject to a data-use agreement specifying non-commercial research use and no attempt at re-identification. Aggregate materials that carry no re-identification risk, namely the full correlation matrix, the invariance model specifications, the attrition analyses, the complete model syntax, and the robustness-check output, are provided without restriction in the Supplementary Materials.

## Supplementary material

Supplementary Code: Python modules reproducing every analysis reported in the article and in the Supplementary Material.
