# Supplementary material for: Problematic smartphone use and academic procrastination over an academic year: a four-wave random intercept cross-lagged panel study of Chinese undergraduates
Source: Front Psychol. 2026 Oct 6;17:1935647. doi: 10.3389/fpsyg.2026.1935647 (PMC13680411; doi:10.3389/fpsyg.2026.1935647)
Supplement: Supplementary file 1 [file supplementary_file_1.docx]

**Supplementary Material**

Problematic Smartphone Use and Academic Procrastination Over an Academic Year: A Four-Wave Random Intercept Cross-Lagged Panel Study of Chinese Undergraduates

All results below were produced from the analytic dataset of N = 512 participants with the code listed in S4. Estimation used full-information maximum likelihood with robust (sandwich) standard errors under Python 3.11.9 (NumPy 2.4.6, SciPy 1.17.1, pandas 3.0.5). Confidence intervals for unstandardized parameters are Wald intervals, estimate ± 1.96 × SE, on the robust standard error. Where a value in the article and the value here differ in the last reported digit, the difference is rounding.

**S1. Zero-order correlation matrix**

*Table S1a. Correlations among the eight focal composites.*

|  | **1** | **2** | **3** | **4** | **5** | **6** | **7** | **8** |
| --- | --- | --- | --- | --- | --- | --- | --- | --- |
| 1. PSU T1 | — |  |  |  |  |  |  |  |
| 2. PSU T2 | .780** | — |  |  |  |  |  |  |
| 3. PSU T3 | .759** | .830** | — |  |  |  |  |  |
| 4. PSU T4 | .742** | .799** | .823** | — |  |  |  |  |
| 5. AP T1 | .216** | .179** | .178** | .220** | — |  |  |  |
| 6. AP T2 | .249** | .151** | .189** | .218** | .892** | — |  |  |
| 7. AP T3 | .249** | .208** | .215** | .239** | .873** | .896** | — |  |
| 8. AP T4 | .251** | .235** | .270** | .295** | .853** | .873** | .899** | — |

*Note. N ranges from 422 to 512 depending on the pair (pairwise complete). PSU = problematic smartphone use; AP = academic procrastination. * p < .05, ** p < .01.*

*Table S1b. Correlations of the focal composites with the six T1 covariates, and covariate intercorrelations.*

|  | **9** | **10** | **11** | **12** | **13** | **14** |
| --- | --- | --- | --- | --- | --- | --- |
| 1. PSU T1 | .084 | .036 | .010 | −.060 | −.165** | .217** |
| 2. PSU T2 | .071 | −.021 | −.009 | −.049 | −.122** | .185** |
| 3. PSU T3 | .095* | −.010 | −.015 | −.115* | −.165** | .152** |
| 4. PSU T4 | .111* | .008 | −.052 | −.091 | −.103* | .188** |
| 5. AP T1 | .049 | .004 | .042 | −.104* | −.156** | .095* |
| 6. AP T2 | .062 | .017 | .021 | −.093* | −.121** | .035 |
| 7. AP T3 | .048 | .019 | .016 | −.097* | −.138** | .058 |
| 8. AP T4 | .082 | .041 | .028 | −.087 | −.172** | .046 |
| 9. Gender | — | −.060 | .011 | .009 | −.035 | .070 |
| 10. Only-child status |  | — | −.034 | .029 | .036 | .059 |
| 11. Grade |  |  | — | .020 | −.074 | .008 |
| 12. Study hours |  |  |  | — | .024 | −.026 |
| 13. Self-control |  |  |  |  | — | −.244** |
| 14. Negative affect |  |  |  |  |  | — |

*Note. Covariates were measured once, at T1, and are complete for all 512 participants. Gender coded 0 = female, 1 = male; only-child status 0 = no, 1 = yes. * p < .05, ** p < .01.*

Across the sixteen PSU–AP wave pairings the correlations range from .151 to .295. PSU and AP correlate with trait self-control between −.172 and −.103, and with trait negative affect between .035 and .217.

**S2. Longitudinal measurement invariance and the item-reduced sensitivity check**

Invariance was tested separately for PSU (10 items) and AP (5 items) across the four waves. Each model specifies one factor per wave, all four factors freely intercorrelated; the first item's loading is fixed to 1 in every wave for scaling; the residual of each item is correlated with the residual of the same item at every other wave; and the latent means are fixed at zero throughout. The configural model frees loadings and intercepts by wave, the metric model constrains loadings equal across waves, and the scalar model constrains loadings and intercepts equal across waves. Because the latent means are fixed at zero, the scalar step constrains item intercepts and latent means jointly; the row labelled “scalar, latent means free” relaxes that by freeing the latent means at T2 to T4, which is the more common specification, and is reported here so that the two are distinguishable.

| **Model** | **χ²** | **df** | **CFI** | **TLI** | **RMSEA** | **SRMR** | **ΔCFI** |
| --- | --- | --- | --- | --- | --- | --- | --- |
| PSU (10 items) |  |  |  |  |  |  |  |
| Configural | 715.12 | 674 | .996 | .995 | .011 | .026 | — |
| Metric | 767.30 | 701 | .993 | .992 | .014 | .032 | .003 |
| Scalar | 971.01 | 731 | .975 | .973 | .025 | .032 | .018 |
| Scalar, latent means free | 953.91 | 728 | .976 | .975 | .025 | .032 | .017 |
| Partial scalar (item 7 intercept freed) | 940.88 | 728 | .978 | .976 | .024 | .032 | .015 |
| Partial scalar, latent means free | 929.11 | 725 | .979 | .977 | .023 | .032 | .014 |
| AP (5 items) |  |  |  |  |  |  |  |
| Configural | 178.40 | 134 | .995 | .993 | .025 | .014 | — |
| Metric | 186.85 | 146 | .996 | .994 | .023 | .016 | −.000 |
| Scalar | 271.84 | 161 | .988 | .986 | .037 | .016 | .008 |
| Scalar, latent means free | 243.19 | 158 | .991 | .989 | .032 | .016 | .005 |

*Note. ΔCFI for the metric model is relative to the configural model; for every scalar and partial-scalar model it is relative to the metric model. A ΔCFI above .01 is taken as evidence against the added constraint.*

PSU therefore retains metric invariance only. Freeing the least invariant item's intercept (item 7) leaves ΔCFI at .015, and freeing the latent means as well leaves it at .014, so neither relaxation brings PSU within the .01 criterion. AP meets full scalar invariance under both specifications (ΔCFI = .008 and .005).

*Table S2b. Item-reduced PSU composite: correlation between the ten-item composite and each nine-item composite omitting one item.*

| **Composite** | **T1** | **T2** | **T3** | **T4** |
| --- | --- | --- | --- | --- |
| Omitting item 1 | .9942 | .9939 | .9949 | .9948 |
| Omitting item 2 | .9941 | .9946 | .9940 | .9949 |
| Omitting item 3 | .9946 | .9940 | .9942 | .9943 |
| Omitting item 4 | .9941 | .9937 | .9942 | .9954 |
| Omitting item 5 | .9945 | .9946 | .9950 | .9950 |
| Omitting item 6 | .9951 | .9953 | .9951 | .9953 |
| Omitting item 7 | .9940 | .9940 | .9944 | .9947 |
| Omitting item 8 | .9931 | .9936 | .9941 | .9941 |
| Omitting item 9 | .9946 | .9947 | .9947 | .9953 |
| Omitting item 10 | .9943 | .9942 | .9942 | .9954 |

*Note. Item 7 is the item whose intercept was freed in the partial-scalar model. Dropping it changes the composite very little at any wave, which is why the structural estimates are insensitive to that respecification; this is indirect evidence and not a substitute for a latent RI-CLPM.*

**S3. Unstandardized RI-CLPM estimates with robust standard errors**

Every freed parameter of the full RI-CLPM is listed below on the unstandardized metric, with robust standard errors, Wald z, two-sided p, and 95% confidence intervals. Model fit: χ²(45) = 67.72, p = .016, CFI = .994, TLI = .988, RMSEA = .031, 90% CI [.014, .046], SRMR = .017 (.022 against the complete-case covariance matrix). The independence baseline used for CFI and TLI is χ²(91) = 4069.65.

| **Parameter** | **b** | **SE** | **z** | **p** | **95% CI** | **β** |
| --- | --- | --- | --- | --- | --- | --- |
| RI-AP ← gender | .166 | .113 | 1.47 | .142 | [−.055, .387] | .066 |
| RI-AP ← grade | .036 | .049 | 0.74 | .458 | [−.060, .133] | .033 |
| RI-AP ← negative affect | .048 | .085 | 0.57 | .570 | [−.118, .215] | .026 |
| RI-AP ← only-child status | .084 | .113 | 0.74 | .461 | [−.139, .306] | .033 |
| RI-AP ← self-control | −.217 | .073 | −2.95 | .003 | [−.361, −.073] | −.132 |
| RI-AP ← study hours | −.021 | .010 | −2.15 | .032 | [−.039, −.002] | −.098 |
| RI-PSU ← gender | .122 | .076 | 1.60 | .109 | [−.027, .272] | .072 |
| RI-PSU ← grade | −.021 | .035 | −0.60 | .548 | [−.089, .047] | −.028 |
| RI-PSU ← negative affect | .245 | .063 | 3.88 | <.001 | [.121, .368] | .195 |
| RI-PSU ← only-child status | −.008 | .076 | −0.10 | .917 | [−.158, .142] | −.005 |
| RI-PSU ← self-control | −.134 | .057 | −2.37 | .018 | [−.245, −.023] | −.122 |
| RI-PSU ← study hours | −.010 | .006 | −1.52 | .128 | [−.023, .003] | −.070 |
| α1 | −.054 | .144 | −0.37 | .711 | [−.337, .230] | −.061 |
| α2 | .309 | .114 | 2.71 | .007 | [.086, .533] | .273 |
| α3 | .291 | .077 | 3.80 | <.001 | [.141, .441] | .277 |
| β1 | .112 | .134 | 0.83 | .404 | [−.151, .374] | .114 |
| β2 | .164 | .120 | 1.37 | .171 | [−.071, .400] | .158 |
| β3 | .247 | .113 | 2.18 | .029 | [.025, .468] | .228 |
| δ1 | −.231 | .151 | −1.52 | .128 | [−.527, .066] | −.259 |
| δ2 | .130 | .107 | 1.21 | .227 | [−.081, .340] | .126 |
| δ3 | .080 | .083 | 0.97 | .333 | [−.082, .242] | .077 |
| γ1 | .094 | .108 | 0.86 | .388 | [−.119, .306] | .097 |
| γ2 | −.015 | .130 | −0.12 | .906 | [−.270, .239] | −.013 |
| γ3 | .409 | .093 | 4.38 | <.001 | [.226, .592] | .374 |
| RI-AP ↔ RI-AP | 1.527 | .069 | 22.12 | <.001 | [1.392, 1.662] | — |
| RI-PSU ↔ RI-AP | .250 | .051 | 4.92 | <.001 | [.150, .350] | .235 |
| RI-PSU ↔ RI-PSU | .656 | .044 | 14.99 | <.001 | [.570, .742] | — |
| gender ↔ gender | .250 | .001 | 321.61 | <.001 | [.248, .251] | — |
| gender ↔ grade | .006 | .025 | 0.26 | .799 | [−.043, .056] | .011 |
| gender ↔ negative affect | .024 | .015 | 1.59 | .112 | [−.005, .053] | .070 |
| gender ↔ only-child status | −.015 | .011 | −1.35 | .177 | [−.036, .007] | −.060 |
| gender ↔ self-control | −.014 | .017 | −0.80 | .423 | [−.047, .020] | −.035 |
| gender ↔ study hours | .028 | .133 | 0.21 | .832 | [−.232, .288] | .009 |
| grade ↔ grade | 1.300 | .044 | 29.46 | <.001 | [1.214, 1.387] | — |
| grade ↔ negative affect | .006 | .035 | 0.18 | .856 | [−.062, .074] | .008 |
| grade ↔ self-control | −.064 | .039 | −1.65 | .100 | [−.141, .012] | −.074 |
| grade ↔ study hours | .137 | .296 | 0.46 | .644 | [−.443, .717] | .020 |
| negative affect ↔ negative affect | .453 | .022 | 20.30 | <.001 | [.409, .496] | — |
| only-child status ↔ grade | −.019 | .025 | −0.78 | .437 | [−.069, .030] | −.034 |
| only-child status ↔ negative affect | .020 | .015 | 1.33 | .182 | [−.009, .049] | .059 |
| only-child status ↔ only-child status | .248 | .002 | 125.42 | <.001 | [.244, .252] | — |
| only-child status ↔ self-control | .014 | .017 | 0.81 | .417 | [−.019, .047] | .036 |
| only-child status ↔ study hours | .086 | .134 | 0.64 | .521 | [−.176, .347] | .029 |
| self-control ↔ negative affect | −.125 | .024 | −5.34 | <.001 | [−.172, −.079] | −.244 |
| self-control ↔ self-control | .586 | .031 | 19.08 | <.001 | [.526, .647] | — |
| study hours ↔ negative affect | −.104 | .181 | −0.58 | .565 | [−.460, .251] | −.026 |
| study hours ↔ self-control | .110 | .202 | 0.55 | .586 | [−.286, .507] | .024 |
| study hours ↔ study hours | 36.264 | 2.016 | 17.99 | <.001 | [32.313, 40.216] | — |
| wAP1 ↔ wAP1 | .220 | .039 | 5.57 | <.001 | [.142, .297] | — |
| wAP2 ↔ wAP2 | .206 | .029 | 7.03 | <.001 | [.149, .264] | — |
| wAP3 ↔ wAP3 | .222 | .033 | 6.83 | <.001 | [.159, .286] | — |
| wAP4 ↔ wAP4 | .210 | .023 | 9.02 | <.001 | [.164, .255] | — |
| wPSU1 ↔ wAP1 | −.044 | .029 | −1.51 | .130 | [−.100, .013] | −.197 |
| wPSU1 ↔ wPSU1 | .223 | .032 | 7.08 | <.001 | [.161, .285] | — |
| wPSU2 ↔ wAP2 | −.065 | .023 | −2.76 | .006 | [−.111, −.019] | −.338 |
| wPSU2 ↔ wPSU2 | .163 | .036 | 4.54 | <.001 | [.093, .234] | — |
| wPSU3 ↔ wAP3 | .033 | .018 | 1.78 | .075 | [−.003, .069] | .145 |
| wPSU3 ↔ wPSU3 | .209 | .022 | 9.66 | <.001 | [.167, .252] | — |
| wPSU4 ↔ wAP4 | .047 | .017 | 2.70 | .007 | [.013, .080] | .181 |
| wPSU4 ↔ wPSU4 | .225 | .023 | 9.81 | <.001 | [.180, .269] | — |

*Note. b is unstandardized; β is the fully standardized solution (paths rescaled by the ratio of latent standard deviations, residual covariances divided by the total implied standard deviations of the two components). α = autoregressive PSU, β = autoregressive AP, γ = PSU → AP, δ = AP → PSU. Robust standard errors are 5 to 10 per cent larger than the expected-information standard errors reported in Table 5 of the article; no inference changes.*

Constrained models. Pooling the three γ paths degrades fit, Δχ²(2) = 11.16, p = .004; pooling the three δ paths does not, Δχ²(2) = 4.85, p = .088; pooling both simultaneously degrades fit, Δχ²(4) = 11.92, p = .018. The pooled estimates are γ_avg = .213 (SE = .058, p = <.001) from the γ-only model and δ_avg = −.013 (SE = .074, p = .862) from the δ-only model, both on the standardized metric. Constraining γ3 = δ3 degrades fit, Δχ²(1) = 9.16, p = .002; the corresponding Wald tests give χ²(1) = 10.31, p = .001 on robust and χ²(1) = 9.57, p = .002 on expected-information standard errors.

Random intercepts. The residual variances of the two random intercepts in the six-covariate model are .656 (RI-PSU) and 1.527 (RI-AP), and their residual correlation is .250; the total implied correlation, which equals the unconditional random-intercept correlation, is .277. Relative to the model-implied total variance at each wave, the random intercepts account for between 74.3% and 80.3% of PSU variance and between 85.6% and 88.3% of AP variance.

*Table S3b. Traditional CLPM comparison, with and without the six T1 covariates.*

| **Model / path** | **χ²** | **CFI** | **TLI or β** | **RMSEA or p** |  |
| --- | --- | --- | --- | --- | --- |
| Without covariates (fit) | χ²(12) = 273.25 | .933 | .843 | .206 |  |
| α1 |  |  | .777 | <.001 |  |
| α2 |  |  | .822 | <.001 |  |
| α3 |  |  | .807 | <.001 |  |
| β1 |  |  | .879 | <.001 |  |
| β2 |  |  | .888 | <.001 |  |
| β3 |  |  | .882 | <.001 |  |
| γ1 |  |  | .061 | .003 |  |
| γ2 |  |  | .066 | .002 |  |
| γ3 |  |  | .093 | <.001 |  |
| δ1 |  |  | .013 | .678 |  |
| δ2 |  |  | .058 | .029 |  |
| δ3 |  |  | .078 | .005 |  |
| With covariates (fit) | χ²(12) = 269.53 | .935 | .509 | .205 |  |
| α1 |  |  | .773 | <.001 |  |
| α2 |  |  | .817 | <.001 |  |
| α3 |  |  | .802 | <.001 |  |
| β1 |  |  | .884 | <.001 |  |
| β2 |  |  | .887 | <.001 |  |
| β3 |  |  | .876 | <.001 |  |
| γ1 |  |  | .073 | <.001 |  |
| γ2 |  |  | .065 | .003 |  |
| γ3 |  |  | .088 | <.001 |  |
| δ1 |  |  | .011 | .709 |  |
| δ2 |  |  | .043 | .097 |  |
| δ3 |  |  | .081 | .003 |  |

*Note. For the fit rows the last three columns are CFI, TLI and RMSEA; for the path rows they are the standardized coefficient and its p value. Autoregressive paths exceed .77 in both models. Five of the six cross-lagged paths are significant in each; the first-interval AP → PSU path is not (p = .678 without covariates, p = .709 with them).*

Composite-level common-method-factor model. Adding a method factor loading on all eight wave-level composites to the jointly pooled model improves fit, Δχ²(8) = 22.34, p = .004, so a common-method component is present. The specification is nevertheless only weakly identified at eight indicators. With the method-factor variance fixed at one to remove the scale indeterminacy, the pooled paths move from γ_avg = .205 and δ_avg = .047 without the factor to .101 and .459 with it, and the method-factor loadings return standard errors between 0.13 and 3.34 times their point estimates. The random-intercept covariance under this specification is .291 (SE = .056). These estimates are reported for completeness; they do not support an inference in either direction about whether the cross-lagged or the trait-level estimates are inflated by shared method variance.

**S4. Model specifications and analysis code**

The analyses were re-run from the raw analytic dataset for this supplement. The code accompanies this file as a set of Python modules: sem.py (a RAM-form structural equation model with full-information maximum likelihood, analytic gradients, robust sandwich standard errors and the usual fit indices), models.py (the model builders described below), run_invariance.py (S2), run_misc.py (S1 and S5), collect.py (S3), run_power.py (S6) and run_r3.py (S3c, S7 and S8). Software versions: Python 3.11.9, NumPy 2.4.6, SciPy 1.17.1, pandas 3.0.5.

**Full RI-CLPM (Table 5 of the article)**

RI-PSU and RI-AP are random intercepts with loadings fixed to 1 on all four wave indicators of their construct. wPSU1 to wPSU4 and wAP1 to wAP4 are within-person components, each with a loading fixed to 1 on its own wave indicator. Indicator residual variances are fixed to 0, so the composites are treated as measured without error. Within-person components at wave t+1 are regressed on both components at wave t: wPSU(t+1) on wPSU(t) (αt) and wAP(t) (δt); wAP(t+1) on wAP(t) (βt) and wPSU(t) (γt). wPSU1 and wAP1 have free variances and a free covariance; wPSU2 to wPSU4 and wAP2 to wAP4 have free residual variances and free within-wave residual covariances. RI-PSU and RI-AP have free variances and a free covariance and are each regressed on all six T1 covariates, which form a saturated exogenous block. Random intercepts are constrained uncorrelated with every within-person component. Observed means are freely estimated, so the mean structure is saturated. Free parameters: 60 in the covariance structure plus 14 means; df = 45.

**Stationarity-constrained models (Table 1)**

Three constrained versions of the full model: γ1 = γ2 = γ3 with the δ paths free (58 covariance-structure parameters); δ1 = δ2 = δ3 with the γ paths free (58); and both sets pooled simultaneously (56). Each is compared with the full model by likelihood-ratio test on 2, 2 and 4 degrees of freedom.

**Equality-constraint test of the third-interval asymmetry (Table 2)**

The full model refitted with γ3 and δ3 forced to a single parameter (59 covariance-structure parameters), compared with the full model on 1 degree of freedom. Wald versions of the same contrast use the robust and the expected-information covariance matrices of the two free estimates.

**Traditional CLPM comparison (Section 4.4)**

The eight observed composites are regressed directly, wave on preceding wave, with the same four paths per interval and no random intercepts. The covariate-matched version regresses all six T1 covariates on all eight occasions, the CLPM analogue of regressing them on both random intercepts.

**Common-method-factor models (Sections 3.4 and 4.4)**

At the item level, a single unmeasured method factor is added to a T1-only confirmatory factor analysis of all 15 focal items. At the composite level, a method factor loading freely on all eight wave-level composites is added to the jointly pooled RI-CLPM; its variance is fixed at one to remove the scale indeterminacy between the loadings and the factor variance.

**Longitudinal invariance models (S2)**

As described in S2: one factor per wave, all pairwise factor covariances free, marker-variable identification, residuals of the same item correlated across all wave pairs, latent means fixed at zero, and loadings and then intercepts constrained equal across waves.

**S5. Attrition analyses and the auxiliary-variable specification**

Attrition was strictly monotone: no participant who missed a wave returned at a later one, so the observed data reduce to four response patterns.

| **Response pattern** | **Waves observed** | **n** | **%** |
| --- | --- | --- | --- |
| Complete at all four waves | 1111 | 422 | 82.4% |
| T1 through T3 only | 1110 | 14 | 2.7% |
| T1 and T2 only | 1100 | 24 | 4.7% |
| T1 only | 1000 | 52 | 10.2% |
| Total enrolled |  | 512 | 100.0% |

*Note. Wave-level sample sizes are therefore 512, 460, 436 and 422.*

Little's test of missingness completely at random, computed on the eight focal composites, does not reject the null, χ²(12) = 9.20, p = .686. On all fourteen model variables it also does not reject, χ²(30) = 37.65, p = .159. A non-significant Little's test is not, however, evidence that missingness is unrelated to observed variables, and the completer-versus-attriter comparisons below show that it is.

| **T1 variable** | **Completers (n = 422)** | **Attriters (n = 90)** | **t** | **df** | **p** |
| --- | --- | --- | --- | --- | --- |
| T1 study hours | 18.32 (6.01) | 16.75 (5.95) | 2.26 | 130.65 | .025 |
| T1 negative affect | 1.46 (0.67) | 1.68 (0.67) | −2.88 | 129.48 | .005 |
| T1 self-control | 3.00 (0.78) | 2.94 (0.72) | 0.68 | 136.49 | .497 |
| T1 PSU | 2.98 (0.95) | 3.09 (1.02) | −0.94 | 124.22 | .351 |
| T1 AP | 2.85 (1.32) | 2.93 (1.42) | −0.47 | 124.03 | .642 |
| Gender | 0.52 (0.50) | 0.51 (0.50) | 0.13 | 129.34 | .893 |
| Only-child status | 0.45 (0.50) | 0.49 (0.50) | −0.70 | 128.94 | .483 |
| Grade | 2.54 (1.13) | 2.47 (1.18) | 0.51 | 126.29 | .614 |

*Note. Welch two-sample t tests. Means with standard deviations in parentheses. Completers are participants observed at T4.*

Attriters reported fewer T1 study hours and higher T1 negative affect; the remaining six comparisons are not significant. Those two variables were therefore entered as auxiliary variables in the estimation using the saturated-correlates approach (Graham, 2003): each auxiliary is freely correlated with the other auxiliary, with every observed model variable, and with the residuals of the endogenous variables, and is assigned no structural path, so it informs estimation of the missing-data mechanism without altering the hypothesized model. Because these two variables predict dropout, missingness is not completely at random; missingness at random concerns the unobserved values and is not testable from these data.

**S6. Monte Carlo power simulation**

Power for the two pooled cross-lagged paths was estimated by simulating 200 four-wave datasets at N = 512 under the population values stated in the Method section, drawing each wave's parameter independently from its stated range: autoregressive PSU .460 to .580, autoregressive AP .470 to .540, γ (PSU → AP) .360 to .420, and δ (AP → PSU) .150 to .210. Between-person variances were set to .656 (RI-PSU) and 1.527 (RI-AP) with a covariance of .250; within-person T1 variances to .223 and .220 with a covariance of −.044; and within-person residual variances at each later wave were set so that total within-person variance stays constant across waves. The realised monotone attrition pattern (512, 460, 436, 422) was imposed on every replication. Each dataset was fitted with the jointly pooled RI-CLPM and the pooled paths tested at α = .05 on robust standard errors.

| **Pooled path** | **Mean estimate** | **SD of estimates** | **Mean z** | **Empirical power** |
| --- | --- | --- | --- | --- |
| γ_avg (H1) | 0.385 | 0.055 | 7.40 | 1.000 |
| δ_avg (H2) | 0.180 | 0.064 | 2.93 | 0.805 |

*Note. 200 of 200 replications converged. Unstandardized metric. Empirical power is the proportion of replications in which the pooled path was significant at α = .05, two-tailed.*

The design is therefore very well powered for an effect the size hypothesized for H1 and adequately powered for one the size hypothesized for H2. Power against effects appreciably smaller than the hypothesized δ is correspondingly lower: with the sampling standard deviation of 0.064 obtained here, a true δ of .10 would be detected in roughly one replication in three.

*Table S3c. Standardized coefficients with delta-method standard errors, compared with a linear rescaling of the unstandardized standard error.*

| **Parameter** | **β** | **SE (delta)** | **SE (rescaled)** | **z** | **p** | **95% CI** |
| --- | --- | --- | --- | --- | --- | --- |
| wPSU1 → wPSU2 (α1) | −.061 | .164 | .163 | −0.37 | .711 | [−.38, .26] |
| wPSU2 → wPSU3 (α2) | .273 | .098 | .101 | 2.79 | .005 | [.08, .46] |
| wPSU3 → wPSU4 (α3) | .277 | .074 | .073 | 3.77 | <.001 | [.13, .42] |
| wAP1 → wAP2 (β1) | .114 | .137 | .137 | 0.83 | .406 | [−.16, .38] |
| wAP2 → wAP3 (β2) | .158 | .111 | .115 | 1.42 | .157 | [−.06, .38] |
| wAP3 → wAP4 (β3) | .228 | .108 | .104 | 2.11 | .034 | [.02, .44] |
| wPSU1 → wAP2 (γ1) | .097 | .111 | .112 | 0.87 | .384 | [−.12, .31] |
| wPSU2 → wAP3 (γ2) | −.013 | .113 | .113 | −0.12 | .906 | [−.24, .21] |
| wPSU3 → wAP4 (γ3) | .374 | .080 | .085 | 4.68 | <.001 | [.22, .53] |
| wAP1 → wPSU2 (δ1) | −.259 | .168 | .170 | −1.54 | .123 | [−.59, .07] |
| wAP2 → wPSU3 (δ2) | .126 | .101 | .104 | 1.24 | .213 | [−.07, .32] |
| wAP3 → wPSU4 (δ3) | .077 | .079 | .080 | 0.97 | .331 | [−.08, .23] |
| wPSU1 ↔ wAP1 | −.197 | .139 | — | −1.42 | .157 | [−.47, .08] |
| wPSU2 ↔ wAP2 | −.338 | .137 | — | −2.47 | .014 | [−.61, −.07] |
| wPSU3 ↔ wAP3 | .145 | .081 | — | 1.79 | .073 | [−.01, .30] |
| wPSU4 ↔ wAP4 | .181 | .070 | — | 2.57 | .010 | [.04, .32] |
| RI-PSU ↔ RI-AP (zero-order) | .277 | .048 | — | 5.74 | <.001 | [.18, .37] |
| RI-PSU ↔ RI-AP (adjusted) | .250 | .049 | — | 5.08 | <.001 | [.15, .35] |
| Pooled γ_avg | .213 | .066 | .058 | 3.22 | .001 | [.08, .34] |
| Pooled δ_avg | −.013 | .075 | .074 | −0.17 | .863 | [−.16, .13] |

*Note. The delta-method standard error differentiates the standardized quantity with respect to the full free-parameter vector and combines that gradient with the robust sandwich covariance matrix, so the uncertainty in the variance parameters entering the standardization is carried through. The rescaled column multiplies the unstandardized standard error by the ratio of the standardized to the unstandardized point estimate, which treats those variances as fixed. The two agree closely for the autoregressive and cross-lagged paths and differ more for the pooled paths; no inference changes. Table 5 of the article reports the delta-method column.*

**S7. RI-CLPM refitted on an item-reduced PSU composite**

The partial-scalar model in S2 frees the intercept of PSU item 7. Because a composite that correlates almost perfectly with another need not yield the same structural estimates, the full RI-CLPM was refitted with PSU scored from the remaining nine items, holding everything else fixed. Fit is equivalent to the ten-item model: χ²(45) = 66.51, p = .020, CFI = .995, TLI = .989, RMSEA = .031, SRMR = .017. The focal estimates are unchanged to within .02.

| **Parameter** | **β (10 items)** | **β (9 items)** | **Difference** | **SE (9 items)** | **p (9 items)** |
| --- | --- | --- | --- | --- | --- |
| wPSU1 → wPSU2 (α1) | −.061 | −.062 | −.002 | .141 | .659 |
| wPSU2 → wPSU3 (α2) | .273 | .215 | −.059 | .105 | .041 |
| wPSU3 → wPSU4 (α3) | .277 | .228 | −.050 | .083 | .006 |
| wAP1 → wAP2 (β1) | .114 | .118 | .004 | .135 | .383 |
| wAP2 → wAP3 (β2) | .158 | .167 | .010 | .110 | .129 |
| wAP3 → wAP4 (β3) | .228 | .227 | −.000 | .107 | .034 |
| wPSU1 → wAP2 (γ1) | .097 | .113 | .016 | .104 | .275 |
| wPSU2 → wAP3 (γ2) | −.013 | −.010 | .004 | .119 | .934 |
| wPSU3 → wAP4 (γ3) | .374 | .378 | .005 | .083 | <.001 |
| wAP1 → wPSU2 (δ1) | −.259 | −.238 | .020 | .156 | .126 |
| wAP2 → wPSU3 (δ2) | .126 | .140 | .014 | .104 | .176 |
| wAP3 → wPSU4 (δ3) | .077 | .058 | −.019 | .086 | .504 |
| RI-PSU ↔ RI-AP (adjusted) | .250 | .253 | .003 | .048 |  |

*Note. Standardized solution, delta-method standard errors. The nine-item composite omits item 7.*

The constrained-model tests reach the same conclusions on the nine-item composite as on the ten-item one: pooling the three γ paths is rejected, Δχ²(2) = 10.80, p = .005; pooling the three δ paths is not, Δχ²(2) = 4.29, p = .117; and constraining γ3 = δ3 degrades fit, Δχ²(1) = 10.76, p = .001. The scalar-invariance failure for PSU therefore does not appear to be driving the structural results. This is still a composite-level check: it does not substitute for a latent RI-CLPM carrying partial-invariance constraints, which this sample size does not support.

**S8. Design-effect sensitivity to unmodelled clustering**

Course-section identifiers were used to administer the survey but were not carried into the analytic dataset, so no cluster-robust correction is possible. The consequence can still be bounded arithmetically. Recruitment covered 14 sections, giving a mean cluster size of about 37, so a design effect of 1 + (m − 1)ρ inflates every standard error by the square root of that quantity. The table applies this to the T3→T4 γ path, which carries the article's central claim, across a range of intraclass correlations.

| **ρ** | **Design effect** | **SE inflation** | **SE** | **z** | **p** | **95% CI** |
| --- | --- | --- | --- | --- | --- | --- |
| .00 | 1.00 | 1.00 | .080 | 4.68 | <.001 | [.22, .53] |
| .01 | 1.36 | 1.16 | .093 | 4.02 | <.001 | [.19, .56] |
| .02 | 1.71 | 1.31 | .105 | 3.57 | <.001 | [.17, .58] |
| .05 | 2.78 | 1.67 | .133 | 2.80 | .005 | [.11, .64] |
| .10 | 4.56 | 2.13 | .171 | 2.19 | .029 | [.04, .71] |
| .15 | 6.34 | 2.52 | .201 | 1.86 | .063 | [−.02, .77] |
| .20 | 8.11 | 2.85 | .228 | 1.64 | .101 | [−.07, .82] |

*Note. ρ is the assumed intraclass correlation within course sections. The unadjusted standard error, at ρ = 0, is the delta-method value reported in Table 5 of the article. β = .374 throughout.*

The path loses significance at ρ ≈ .13, a design effect of about 5.7 and an inflation of the standard error by a factor of about 2.4. An intraclass correlation of that size would be unusual for course sections within a single institution, so the central estimate is unlikely to rest on clustering alone. The calculation nevertheless assumes one common design effect across all parameters, takes the point estimate as unaffected by clustering, and is not a substitute for the cluster-robust or multilevel treatment the data do not permit.

**Reference**

Graham, J. W. (2003). Adding missing-data-relevant variables to FIML-based structural equation models. Structural Equation Modeling: A Multidisciplinary Journal, 10, 80–100.
